# Supplementary material for: Improving the Detection, Assessment, Management and Prevention of Delirium in Hospices (the DAMPen-D study): protocol for a co-design and feasibility study of a flexible and scalable implementation strategy to deliver guideline-adherent delirium care
Source: BMJ Open. 2022 Jul 13;12(7):e060450. doi: 10.1136/bmjopen-2021-060450 (PMC9280901; doi:10.1136/bmjopen-2021-060450)
Supplement: Supplementary data [file bmjopen-2021-060450supp001.pdf]

Online supplemental file 1
'Improving the Detection, Assessment, Management, and Prevention of Delirium in Hospices (the DAMPen-D study): Protocol for a co-design and feasibility study of a flexible and scalable implementation strategy to deliver guideline-adherent delirium care.'

**Case Number_Pre**	Non-identifiable ID number	N/A	Alphnumeric
**Age_Pre**	Patient age	Integer	Years
**Sex_Pre**	Patient gender	Categorical	Male, female, other
**Diagnosis_Pre**	Patient diagnosis	String	Cancer etc.
**Ethnicity_pre**	Patient ethnicity	Categorical	Black, white etc.
**IMD score_Pre**	Postcode converted	Float	
**Adm_Ac_conf_state_Pre**	Evidence of acute confusional state on admission	Binary	Yes/No
**Adm_screen_Pre**	Patient screened for delirium on admission	Binary	Yes/No
**Adm_Screen_by_Pre**	If screened, who completed screening	Categorical	Doctor, Nurse practitioner, Registered Nurse, Other (specify)
**Adm_screen_type_Pre**	If screened, name of screening tool	Alphanumeri c	4AT etc.
**Adm_screen_result_Pre**	If screened, result	Binary	Positive/Negative
**Adm_no_screen_just-Pre**	Was justification given for not screening	Binary	Yes/No
**Adm_no-screen_just-verbatim-Pre**	Was justification given for not screening	String	Verbatim text

Pearson M, *et al. BMJ Open* 2022; 12:e060450. doi: 10.1136/bmjopen-2021-060450

Online supplemental file 1
'Improving the Detection, Assessment, Management, and Prevention of Delirium in Hospices (the DAMPen-D study): Protocol for a co-design and feasibility study of a flexible and scalable implementation strategy to deliver guideline-adherent delirium care.'

**Adm_risk-ass-Pre**	If screening negative or not done was risk assessment carried out?	Binary	Yes/No
**Adm-risk-ass_result_Pre**	If risk assessment carried completed results	Binary	Positive/Negative
**Adm_prev_meas_Pre**	If risk assessment positive were preventive measures put in place	Binary	Yes/No
**Adm_Judge_rationale_pre**	If researcher judgement was required for any of above, give rationale	String	Free text
**Dur_adm_Ac_conf_state_pre**	Evidence of acute confusional state during admission	Binary	Yes/No
**Dur_adm_ Multi_ep_cog_dys_Pre**	Multiple episodes of cognitive dysfunction during admission	Binary	Yes/No
**Dur_adm_ Multi_ep_cog_dys_no_Pre**	Multiple episodes of cognitive dysfunction during admission	Integer	1,2,3

Pearson M, *et al. BMJ Open* 2022; 12:e060450. doi: 10.1136/bmjopen-2021-060450

Online supplemental file 1
'Improving the Detection, Assessment, Management, and Prevention of Delirium in Hospices (the DAMPen-D study): Protocol for a co-design and feasibility study of a flexible and scalable implementation strategy to deliver guideline-adherent delirium care.'

**Dur_adm_screen_Pre**	If patient had multiple episodes was the patient screened	Binary	Yes/No
**Dur_adm_screen_type_Pre**	If screened, name of tool	Alphanumeri c	4AT etc.
**Dur_Adm_Screen_result_Pre**	Result of screening during admission	Binary	Positive/negative
**Dur_adm_Sceen_by_Pre**	Who completed screening during admission	Categorical	Doctor, Nurse practitioner, Registered Nurse, Other (specify)
**Dur_adm_Judge_rationale_Pre**	If researcher judgement was required for any of above, give rationale	String	Verbatim text
**Case_rec_ac_conf_reported_by_Pr e**	Who reported the first episode of acute confusion in the case record	Categorical	Doctor, Nurse practitioner, Registered Nurse, Other (specify)
**Case_rec_date_first_ep_Pre**	Date of first episode of acute confusion in the case record	Date	10.10.2021
**Case_rec_time_first_ep_Pre**	Time of first episode of acute confusion in case record	Time	24hr format

Pearson M, *et al. BMJ Open* 2022; 12:e060450. doi: 10.1136/bmjopen-2021-060450

Online supplemental file 1
'Improving the Detection, Assessment, Management, and Prevention of Delirium in Hospices (the DAMPen-D study): Protocol for a co-design and feasibility study of a flexible and scalable implementation strategy to deliver guideline-adherent delirium care.'

**Case_rec_verbatim_ref_ac_conf_Pre**	Describe each reference to acute confusion in the case record	String	Verbatim text
**Case_rec_ac_conf_tot_days_Pre**	Total duration of acute confusion in days as determined by all the references in the case record	Integer	5 (days) or 0 days if none
**Case_rec_Improve_revers_Pre**	Any evidence of improvement or reversibility of acute confusion during the stay	Categorical	Yes/No/Unsure
**Case_rec_ev_descr_pre**	Describe evidence of reversibility	String	Free text
**Case_rec_Del_present_Pre**	Delirium present	Categorical	Yes/No
**Case_rec_subtype_Pre**	If delirium present what subtype	Categorical	Hypo/Hyper/Mixed
**Case_rec_del_med_ass_Pre**	Medical assessment (DSM-V delirium assessment) to assess for delirium	Binary	Yes/No
**Case_rec_diag_doc_Pre**	Diagnosis of delirium recorded	Categorical	Yes/No

Online supplemental file 1
'Improving the Detection, Assessment, Management, and Prevention of Delirium in Hospices (the DAMPen-D study): Protocol for a co-design and feasibility study of a flexible and scalable implementation strategy to deliver guideline-adherent delirium care.'

**Case_rec_judge_rationale_Pre**	If researcher judgement was required for any of above, give rationale	String	Free text
**Invest_del_ ass_rev_cause_Pre**	Assessment for reversible causes of delirium	Binary	Yes/No
**Invest_med_rev_Pre**	Was a medication review conducted	Binary	Yes/No
**Invest_rev_cause_treat_Pre**	Was a treatment instigated for a reversible cause of delirium	Binary	Yes/No
**Invest_ judge_rationale_Pre**	If researcher judgement was required for any of above, give rationale	String	Free text
**Del_care_plan_Pre**	Delirium care plan documented	Binary	Yes/no
**Del_sev_Pre**	Was delirium severity assessed	Categorical	RASS-PAL + hallucination,RASS -PAL only, hallucination only, other specify, No

Pearson M, *et al. BMJ Open* 2022; 12:e060450. doi: 10.1136/bmjopen-2021-060450

Online supplemental file 1
'Improving the Detection, Assessment, Management, and Prevention of Delirium in Hospices (the DAMPen-D study): Protocol for a co-design and feasibility study of a flexible and scalable implementation strategy to deliver guideline-adherent delirium care.'

**Harm_distress_behaviour_Pre**	Did patient display behaviours harmful or distressing to self or others	Binary	Yes/No
**Sedative_admin_during_del_Pre**	Was sedative administered during period of delirium	Binary	Yes/No
**Sedative_med_type**	Sedative medication type	String	Name of medication
**Sed_ind_Pre**	Sedative medication administered for	Categorical	Delirium, anxiety, breathlessness, nausea, terminal agitation, other, unclear
**Del_risk_discuss_patient_fam**	Was delirium risk and prevention discussed with patients and families of patients without delirium on admission	Categorical	Yes/No/unable
**Del_ep_discuss_patient_Pre**	Was episode of delirium discussed with the patient	Categorical	Yes/No/Unable

Pearson M, et al. BMJ Open 2022; 12:e060450. doi: 10.1136/bmjopen-2021-060450

Online supplemental file 1
‘Improving the Detection, Assessment, Management, and Prevention of Delirium in Hospices (the DAMPen-D study): Protocol for a co-design and feasibility study of a flexible and scalable implementation strategy to deliver guideline-adherent delirium care.’

**Del_ep_discuss_patient_family_Pre**	Was episode of delirium discussed with the patient's family	Categorical	Yes/No/Unable
**Del_info_Pre**	Was any written information about delirium provided to patient or family	Categorical	Yes/No/Unable

Pearson M, *et al. BMJ Open* 2022; 12:e060450. doi: 10.1136/bmjopen-2021-060450
