# Supplementary material for: Improving the Detection, Assessment, Management and Prevention of Delirium in Hospices (the DAMPen-D study): protocol for a co-design and feasibility study of a flexible and scalable implementation strategy to deliver guideline-adherent delirium care
Source: BMJ Open. 2022 Jul 13;12(7):e060450. doi: 10.1136/bmjopen-2021-060450 (PMC9280901; doi:10.1136/bmjopen-2021-060450)
Supplement: Author's manuscript [file bmjopen-2021-060450.draft_revisions.pdf]

BMJ Open is committed to open peer review. As part of this commitment we make the peer review history of every article we publish publicly available.

When an article is published we post the peer reviewers' comments and the authors' responses online. We also post the versions of the paper that were used during peer review. These are the versions that the peer review comments apply to.

The versions of the paper that follow are the versions that were submitted during the peer review process. They are not the versions of record or the final published versions. They should not be cited or distributed as the published version of this manuscript.

BMJ Open is an open access journal and the full, final, typeset and author-corrected version of record of the manuscript is available on our site with no access controls, subscription charges or pay-per-view fees (http://bmjopen.bmj.com).

If you have any questions on BMJ Open's open peer review process please email info.bmjopen@bmj.com

BMJ Open

Improving the Detection, Assessment, Management, and Prevention of Delirium in Hospices (the DAMPen-D study): protocol for a co-design and feasibility study of a flexible and scalable implementation strategy to deliver guideline-adherent delirium care.

Journal:	*BMJ Open*
Manuscript ID	bmjopen-2021-060450
Article Type:	Protocol
Date Submitted by the Author:	22-Dec-2021

Complete List of Authors:	Pearson, Mark; Hull York Medical School, Wolfson Palliative Care Research Centre<br>Jackson, Gillian; Hull York Medical School, Wolfson Palliative Care Research Centre<br>Jackson, Catriona; St James's University Hospital<br>Boland, Jason; Hull York Medical School,<br>Featherstone, Imogen; University of York, Department of Health Sciences<br>Huang, Chao; University of Hull<br>Ogden , Margaret; University of Stirling, Faculty of Social Sciences<br>Sartain, Kathryn; University of Hull; York and Scarborough Teaching Hospitals NHS Foundation Trust<br>Siddiqi, Najma; University of York, Psychiatry, Hull York Medical School, York and Bradford District Care NHS Foundation Trust, Bradford, UK; Research<br>Twiddy, Maureen; University of Hull<br>Johnson, Miriam; The University of Hull, Hull York Medical School

Keywords:	PALLIATIVE CARE, Delirium & cognitive disorders < PSYCHIATRY, Protocols & guidelines < HEALTH SERVICES ADMINISTRATION & MANAGEMENT

SCHOLARONE™
Manuscripts

Improving the Detection, Assessment, Management, and Prevention of Delirium in Hospices (the DAMPen-D study):

Protocol for a co-design and feasibility study of a flexible and scalable implementation strategy to deliver guideline-adherent delirium care.

Pearson, M.*[1], Jackson, G.[1], Jackson, C.[1,2], Boland, J.[1], Featherstone, I.[3], Huang, C.[4], Ogden, M.[5], Sartain, K.[6], Siddiqi, N.[3], Twiddy, M.[4] & Johnson, M.J[1]

* corresponding author: Dr Mark Pearson, Wolfson Palliative Care Research Centre, Hull York Medical School, Allam Medical Building, University of Hull, Hull, HU6 7RX, UK. Tel.: +44 1482 463335

[1] Wolfson Palliative Care Research Centre, Hull York Medical School, University of Hull, Hull, HU6 7RX, UK

[2] Leeds Teaching Hospitals NHS Trust, St James's University Hospital, Leeds, LS9 7TF, UK

[3] Department of Health Sciences, University of York, York, YO10 5DD, UK

[4] Institute of Clinical & Applied Health Research, Hull York Medical School, University of Hull, Hull, HU6 7RX, UK

[5] Public involvement member

[6] York and Scarborough Teaching Hospitals NHS Foundation Trust, York Hospital, Wigginton Road, York, YO31 8HE, UK

Author email addresses:
Pearson, M. mark.pearson@hyms.ac.uk
Jackson, G. gillian.jackson@hyms.ac.uk
Jackson, C. catriona.jackson@doctors.org.uk
Boland, J. jason.boland@hyms.ac.uk
Featherstone, I. imogen.featherstone@york.ac.uk
Huang, C. chao.huang@hyms.ac.uk
Ogden, M. margaretogden@hotmail.com
Sartain, K. kathryn.sartain@york.nhs.uk
Siddiqi, N. najma.siddiqi@york.ac.uk
Twiddy, M. maureen.twiddy@hyms.ac.uk
Johnson, M.J miriam.johnson@hyms.ac.uk

Word count (excluding title page, abstract, references, figures and tables): 3743

For peer review only

Abstract

Introduction Delirium is a complex condition in which altered mental state and cognition causes severe distress and poor clinical outcomes for patients and families, anxiety and stress for the health professionals and support staff providing care, and higher care costs. Hospice patients are at high risk of developing delirium as they frequently have multiple risk factors such as multiple medications, metabolic disturbance, pain, poor sleep, infection and dehydration. Whilst the importance of identifying, preventing and managing delirium is recognised, in practice there is significant variation in care delivery. The primary objective of this study is to inform a future quasi-experimental multi-site comparative evaluation by demonstrating the feasibility of an implementation strategy (designed to help deliver good practice delirium guidelines), participant recruitment, and data collection.

Methods and analysis Three work packages in three hospices in the United Kingdom with public involvement in co-design, study management and stakeholder groups: 1) Experience-Based Co-Design to adapt an existing theoretically-informed implementation strategy (*Creating Learning Environments for Compassionate Care (CLECC)*) to implement delirium guidelines in hospices. 2) Feasibility study to explore ability to collect clinical record data, explanatory process data, and cost data. 3) Realist Process Evaluation to assess the acceptability and flexibility of the implementation strategy. Descriptive statistics, rapid thematic analysis, and a realist logic of analysis will be used be used to analyse quantitative and qualitative data, as appropriate.

Ethics and dissemination Ethical approval for the study has been obtained. A results paper will be submitted to an open access peer-reviewed journal and abstracts submitted to national and international conferences. A lay summary of results will be shared with study site staff and stakeholders.

Study registration: ISRCTN 55416525

Keywords: Delirium; palliative care; guideline implementation; co-design; feasibility; realist process evaluation

Article summary - Strength and limitations of this study:

- Innovative collaborative adaptation of a theoretically-informed implementation strategy (CLECC) to deliver guideline-adherent delirium care in hospices (CLECC-Pal).
- Research waste minimised and patient/carer burden eliminated through use of existing patient outcome and process data.
- Use of implementation theory to investigate how the implementation strategy functions and may be operationalised differently to achieve desired outcomes
- Involvement of public members since study inception and throughout study delivery and management.
- Evaluation of feasibility and acceptability of an implementation strategy before testing at scale.

For peer review only

INTRODUCTION

Delirium is a complex condition characterised by fluctuating impairment of awareness, attention, and cognition.(1) Delirium causes severe distress for patients and families(2), anxiety and stress for the health professionals and support staff providing care, poor clinical outcomes,(3, 4) and higher care costs (e.g. longer inpatient stays).(5, 6) People nearing the end of life have a high risk of delirium,(2) with risk factors such as medication, metabolic disturbance, pain, poor sleep, infection and dehydration acting cumulatively.(7) Effective delirium care is driven by prevention where possible, timely detection and non-pharmacological management, with pharmacological interventions if appropriate.(8, 9)

An international systematic review reported that one-third of people in adult palliative care settings had delirium on admission, with two-thirds developing delirium during the admission.(7) Across health services the health economic impact of delirium is significant. Although data are not available from palliative care settings, other estimates of health service costs from delirium show comparable costs to falls, diabetes and cardiovascular diseases.(10)

NICE Clinical Guideline 103(11) and other international guidelines(12) and standards,(13) recommend strategies for delirium assessment, prevention and management. However, this is difficult in practice, with a disconnection between improved levels of delirium knowledge and the capacity of palliative care practitioners to implement changes. A recent international qualitative systematic review identified that practical and emotional support were needed to enable staff to assess, prevent and manage delirium.(14)

A recent survey of palliative care doctors (n=335) in the United Kingdom found that 38% never used delirium guidelines and that only 13% of palliative care teams used a tool (rather than clinical judgement) to assess for delirium at first inpatient assessment, with even fewer (9%) using a tool on an ongoing basis.(15) Our survey of UK specialist palliative care units (n=220, mostly nurses)(16) found that only 10% ever used a delirium screening tool, with only 5% following NICE guidelines by screening on admission, and only 6% screening daily thereafter. The importance of delirium care has been recognised in a national survey of dying patients, with 92% rating 'being mentally aware' as "very important" and nearly as many (89%) citing 'not being a burden on family'.(17)

Delirium detection, assessment, management and prevention is complex, depending on practical support (screening tools and clinical pathways) and communication (18, 19) between family and friends, volunteers, healthcare assistants (HCAs), nurses, allied health professionals (AHPs), social workers, doctors, hospice managers and board members. It also takes place at some of the most sensitive and emotionally-fraught times in the lives of patients and their families. Therefore, guideline implementation requires a relevant and flexible strategy based on an understanding of how adaptation for different settings can be attained whilst retaining effectiveness.

To address this gap in knowledge about how to implement guideline-adherent delirium care, we shall first adapt an existing theoretically-informed implementation strategy that has been tested in acute hospital wards (*Creating Learning Environments for Compassionate Care (CLECC)*). CLECC has been found to foster and legitimise the reflection, learning, mutual support and innovation that can enable team members to progress from knowing to doing.(20) It comprises a team study day, ward manager action learning sets, peer observations of practice, and involvement of all staff in mid-shift 'cluster discussions' and twice-weekly reflective discussions,(21) and is shown mapped to the TIDieR checklist(22) in Table 1. We will then test the feasibility of a subsequent quasi-experimental study to

For peer review only

evaluate the effect of the adapted CLECC (the intervention) on hospice staff delivery of guideline-adherent delirium care and subsequent improvement in patient outcomes (reduction in the number of delirium days).

Aims and objectives

This study will address three key uncertainties about the implementation of guideline-adherent delirium care in hospices by demonstrating if it is possible to:

- Co-adapt an implementation strategy (Creating Learning Environments for Compassionate Care (CLECC)) for use in hospices (Work Package 1).
- Systematically and reliably collect data (including delirium diagnosis) from clinical records in a way that minimises burden for patients, families, and staff (Work Package 2).
- Collect measures of staff engagement with the implementation strategy, delivery of guideline-adherent delirium care, and the costs of staff involvement (Work Package 2).
- Collect explanatory process data about staff use of the implementation strategy (Work Package 3).
- Estimate the number of hospice sites and in-patient episodes needed for the planned national quasi-experimental study.

METHODS AND ANALYSIS

Design summary

Table 2 presents the research questions and summarises the three Work Packages (WPs) that will enable the above aims and objectives to be met. Figure 1 shows the study timeline and how the work packages are inter-related.

Settings

Three adult hospices in northern England (United Kingdom). Two hospices in this study are located in socio-economically deprived urban areas (one with a significant minority ethnic group population) and one hospice in an affluent rural/urban area. One hospice is run by a national charity, with the other two hospices run by independent charities.

Patient and Public Involvement

This study supports the involvement of patient and public involvement (PPI) in accordance with the framework for good public involvement as detailed by the UK standards for public involvement.(23) Public involvement group members contributed to study design, with one member joining the monthly Study Management Group meetings, co-facilitating workshops (Work Package 1) and a further member Chairing the Study Steering Committee. The study's Public Involvement Group will meet three to four times over the duration of the study to discuss public involvement challenges in the research, the implications of emerging study findings, and the development of public-facing research outputs and the next steps in the research cycle.

For peer review only

Table 1 CLECC(21) components mapped using TIDieR checklist(22)

Component	Why	What	Who	How	Where	When/How much	Tailoring & modifications	Fidelity
**Study day**	Prepare staff for the workplace elements of the intervention	**Procedure:** Introduction to CLECC Activities/discussion Questionnaires Film handouts **Materials:** PowerPoint presentation. Record of attendance. Summary of CLECC leaflet	Appointed hospice lead clinician	Classroom based to include all hospice staff	Comfortable classroom that is geographically separate from the workplace	One day at beginning of implementation period, but may require more than one study day to ensure maximum attendance	Pending Work Package 1 co-design workshops	Attendance and feedback data from hospice lead clinician.
**Action Learning sets**	Real problems from own practice and devise action plan to address	**Procedure:** Session 1: relationships & rules Session 2: valuing staff Session 3: enhancing capacity CLECC Session 4: influencing seniors	Experienced facilitator and 4-8 leads of comparable position	Face to face at hospice site	At hospice site	4 x4 hours action learning sets throughout intervention period	Pending Work Package 1 co-design workshops	Fidelity/ attendance
**Peer review**	Appreciate practice from observer perspective	**Procedure:** 2-3 x 1 hour observations Reflective summary **Materials:** Training video Poster of findings	2 team members nominate or nominated by lead and training given.	Outside of normal role to do this activity	At hospice site	Approximately 30 minute training video prior to commencing 2-3 x 1 hour observations throughout implementation	Pending Work Package 1 co-design workshops	Fidelity
**Mid-shift cluster discussions**	Opportunities for feedback, group problem solving and support to individual team members.	**Procedure:** Mid-shift 5 minute discussion	All team members on shift.	Mid-way through every shift.	At hospice site	5 minute discussion mid-shift, initially instigated by lead but then to be maintained by staff	Pending Work Package 1 co-design workshops	Fidelity
**Reflective discussions**	To prompt personal reflections and narratives about individual experiences	**Procedure:** Scheduled meetings or drop in sessions with planned activities **Materials:** Devise a sustainability plan	All team members, including senior staff and temporary staff.	Can be scheduled time during shift or drop-in sessions.	At hospice site, in a comfortable room on or near place of care.	Number of sessions dependent on the number of subjects needed to be discussed	Pending Work Package 1 co-design workshops	Fidelity

Table 2 Overview of study design

Work Package objective	Research question	Study type	Data collection	Timepoints
1. Refine CLECC-Pal implementation strategy	What are the core and adaptable components of an implementation strategy for guideline-adherent delirium care in hospices?	Experience-based co-design	Workshops	Before and during implementation
2. Demonstrate feasibility of future quasi-experimental study	Is it feasible to collect sufficient outcome data (both implementation and clinical), explanatory process data, and cost data in a future effectiveness evaluative study in palliative care settings?	Feasibility study	Patient demographics and delirium diagnosis & management (clinical records)<br><br>Number of staff engaged in CLECC-Pal	Baseline & follow-up<br><br>During implementation & follow-up
3. Assess acceptability and flexibility of CLECC-Pal implementation strategy	How can a co-designed implementation strategy for guideline-adherent delirium care be operationalised with fidelity to function in different hospice inpatient settings?	Realist process evaluation	Survey<br><br>Fidelity to CLECC-Pal<br><br>Interviews	Baseline & follow-up<br><br>Start, middle & end of 3-month period using CLECC-Pal<br><br>Follow-up

For peer review only

[Insert Figure 1 about here]

Work Package 1: Adaptation (Co-Design) of CLECC for guideline-adherent delirium care

An experience-based co-design (EBCD) group(24-26) of people with lived experience of delirium (themselves or in a family member or friend), staff and management from across the study sites and the region will meet for online workshops (maximum three hours duration) at months 2, 8, and 14 to adapt the CLECC strategy for use in hospices (see **Error! Reference source not found.**). The first of these co-design workshops will be held separately for public and staff to facilitate reflection within a broader public or staff 'group' and to underpin interactions between public and staff at subsequent joint workshops. The interactions in these joint co-design workshops are considered essential for participants to share their (sometimes very different) experiences, develop an appreciation of others' experiences, and open up new ways of thinking about how to meet challenges that will directly inform co-design.(27) Consistent with the INVOLVE principles for co-producing research,(28) workshops will be co-developed with our Public Involvement group and co-facilitated by an experienced Public Involvement group member.

Potential *public* participants will be invited through existing national PPI networks to join the co-design workshops. Potential *hospice staff and management* participants (clinicians, volunteers, managers, and board members) will be invited through existing communication channels at each site and in consultation with managers. Information will be provided for potential participants with an opportunity to discuss in more detail prior to taking part. Workshops will be scheduled to fit with existing commitments and day-to-day practice at each hospice. PPI team member (MO) will provide input into all aspects of invitations, information provision, and workshop design.

We shall endeavour to maximise diversity within the workshops but acknowledge the tension between attaining diversity across every potential aspect and a maximum workable number of workshop participants of around 15. We shall keep this under review with PPI team member MO.

Central to the conduct of the workshops will be the use of 'touch points' to communicate other peoples' experiences and provide a focus to spark discussion and exploration from different perspectives.(25) Touch points are the events which significantly shape people's positive or negative experience of an event or service. It could be the sharing of a personal or professional experience of delirium care by a workshop participant, or a short film or news item about palliative care services generally or delirium specifically. These will be used to trigger discussion about the detection, assessment, prevention, and management of delirium, how CLECC could enable the implementation of delirium guidelines, and how CLECC could be adapted for hospices (CLECC-Pal).

Table 3 provides an overview of the schedule and content of the co-design workshops.

For peer review only

Table 3 Co-design workshops schedule and content

Workshop focus	Participants	When, duration	Content
1a. Introduction and initial refinement of CLECC-Pal	Public members	Month 2<br>2 hours	• Introductions<br>• Discussion about the principles of equitable participation<br>• Discussion about the co-design approach to workshops<br>• Introduction to the CLECC strategy and exploration of priority aspects for adaptation<br>• Identification of individual working groups' role in exploring and refining site- or issue-specific aspects of the CLECC strategy before Workshop 2<br>• Agreement on feedback processes outside of the workshops and focus of agenda for Workshop 2
1b. Introduction and initial refinement of CLECC-Pal	Hospice staff and volunteers	Month 2<br>2 hours	As for Workshop 1a
2. Refinement of CLECC-Pal	Public members, hospice staff and volunteers	Month 8<br>3 hours	• Feedback from individual working groups<br>• Discussion of emerging findings from Work Package 3 (realist process evaluation)<br>• Specification of suggested adaptations to CLECC,<br>• Identification of further individual working groups to refine site- or issue-specific aspects of the CLECC strategy<br>• Agreement on focus of agenda for Workshop 3
3. Final specification of CLECC-Pal and celebration	Public members, hospice staff and volunteers	Month 14<br>3 hours	• Feedback from individual working groups<br>• Discussion of further findings from Work Package 3 (realist process evaluation)<br>• Final specification of adaptations to CLECC<br>• Celebration of co-design outputs

For peer review only

Work package 2: Feasibility study

Feasibility will be assessed in the following key areas:

- Patients:
  - Ability to collect high quality, anonymised delirium outcome and process (extent of guideline-adherent care) data from clinical records
  - Variability of baseline delirium day measures to calculate the sample size for a subsequent national study.
- Staff and volunteers: Number of relevant hospice staff and volunteers' participation in CLECC-Pal activities (proportion of relevant staff engaging and maintaining engagement)
- Economic: Ability to collect cost data in relation to CLECC-Pal staff activities

The co-designed CLECC-Pal (for initial version, see Table 1) will be introduced to clinical and support staff, volunteers, and managers at each hospice in a study day that will include training in guideline-recommended delirium care. The study team will support the identified clinical lead to introduce and use CLECC-Pal, including action learning sets, mid-shift 'cluster discussions', twice-weekly reflective discussions and peer observations of practice, over a minimum 12-week period. The study day ethos will emphasise how hospices should take ownership of using CLECC-Pal with only modest support from the study team.

Data collection and analysis

Patients:

Baseline and follow-up (pre and post) clinical record data will be collected. Data will be collected through remote access to the clinical record where electronic records allow, or from the paper record. Case note collection will comprise:

- Baseline (pre): 50 consecutive patients who completed their in-patient stay immediately prior to the start of the hospice using CLECC-Pal.
- Follow-up (post): 50 consecutive patients completing their in-patient stay from week 4 of starting use of CLECC-Pal.

Clinical record data collected by the researcher will be anonymised at the point of extraction and include:

- Demographic data (baseline only): age, sex, main medical condition, ethnicity, post code (converted to IMD score)
- Delirium diagnosis using the Inouye et al case note tool(29)
- Delirium management: including evidence of use of delirium screening tools, risk assessments and individualised delirium management care plans

Clinical record data will be extracted using an expanded version of the prospectively validated (74% sensitivity, 83% specificity) chart-based instrument developed by Inouye et al. for detecting potential delirium diagnoses from clinical records.(29) The instrument (data extraction pro-forma, see online supplemental file 1) will enable us to assess whether case-note recorded symptoms of delirium can be linked to time-points during the person's admission when actions around delirium assessment, management and prevention (consistent with guidelines) did or did not take place. Our 'expanded' version of the instrument will include questions about other actions to support delirium assessment, management and prevention that may be recorded in the notes, as shown in Table 4. We shall report the percentage of clinical records where information about each of these actions is recorded. Where a person experiences multiple episodes of delirium within one admission, each episode will be recorded separately and linked through the anonymised case number.

For peer review only

Where judgements about what to record on the pro-forma need to be made, justification for these will be recorded on the form. Any uncertainty about how the information in the case-notes should be recorded on the pro-forma will be discussed with a second clinician (CJ) and justification for the final decision recorded.

Table 4 Additional delirium assessment items to be derived from clinical records and means of assessing feasibility of data collection

Delirium-related action	Assessment of feasibility
Use of Richmond Agitation-Sedation Scale and 4AT screening tools	% completed<br>% re-assessments completed at appropriate timepoints
Medication reviews	% completed<br>% re-assessments completed at appropriate timepoints
DSM-V delirium assessment	% completed<br>% re-assessments completed at appropriate timepoints
Degree of sedation or agitation	% completed
Individualised delirium care plans	% completed<br>% reviewed at appropriate timepoints
Presence/absence of delirium	% documenting start and end of delirium episode(s)<br>% documenting delirium-free days

The number of patient records from which it was possible to extract clinical record data *longitudinally* over the duration of their inpatient admission will be reported both as a simple count and as a percentage of the total number of in-patients with a diagnosis of delirium in each hospice each month.

Sample size: Based on our pilot work in one hospice, retrospectively collecting clinical record data for *all* patients whose episode of in-patient care is completed (up to a maximum of 50 per hospice) will provide us with enough data to answer feasibility questions about data quality and enable us to capture frequent events regarding care planning. We do not propose to investigate less-frequent events such as antipsychotic use.

Analysis: Baseline demographic and clinical characteristics of the study population (age, sex, primary medical condition, ethnicity, post code (to derive IMD)) will be presented using descriptive statistics. Mean (SD) will be reported for continuous data and raw count (number, percentage) will be reported for nominal data. The variation around baseline delirium days will be calculated to inform the sample size and number of hospices needed for the subsequent national study.

Staff and volunteers:

In consultation with operational and clinical management at each site, a hospice study lead has been identified through whom the following denominators will be established:

- Number of staff working on or rotating through the in-patient unit of the hospice
- Number of volunteers active within the in-patient unit of the hospice
- Total number of in-patient delirium episodes *or* (if total number cannot be established) number of patients with at least one case-note diagnosis of delirium per in-patient admission in the hospice

Level of staff engagement with CLECC-Pal during the implementation period will be assessed weekly by the hospice study lead completing a rapid report of numbers of:

For peer review only

- staff *indirectly* involved in delivering delirium care who attend the team study day, action learning sets, feedback following peer observations of practice, mid-shift cluster discussions, and reflective discussions
- staff and volunteers who do not engage with CLECC-Pal
- staff and volunteers who decrease or stop their engagement with CLECC-Pal
- peer observations of practice achieved
- people approached, reported by professional group and role, who agree to participate in using CLECC-Pal

The rapid report will also record reasons for:

- staff and volunteers' non-engagement or dropout
- modifications made in the use of CLECC-Pal

Quantitative data will be analysed descriptively using radar plots. Qualitative data will be rapidly analysed deductively using a Framework approach.(30) Analyses will inform more detailed exploration in interviews (WP3) and will be shared with participating hospices to inform their ongoing use of CLECC-Pal.

Economic:

We will assess the feasibility of collecting data about the costs of using CLECC-Pal:

- Number of hours spent by members of staff in CLECC-Pal activities

Work Package 3: Realist Process Evaluation

Critiques of process evaluations have highlighted the importance of methods that can use theory to explore how contexts and mechanisms interact.(31-33) We shall use realist evaluation(34) to capture staff and management insights into how individual-, team-, and organisational-level contexts affect these interactions during implementation,(35) refining Normalisation Process Theory's (NPT) propositions about the mechanisms of coherence, cognitive participation, collective action, and reflexive monitoring.(36-38). Definitions of realist terms used in this Work Package are shown in Table 5. This theoretically-informed understanding of how the implementation strategy *functions*(39) will enable us to explain how hospices may operationalise CLECC-Pal in different ways to achieve the same desired outcomes (for example, by running online learning rather than a team study day, or using self-reflection on practice rather than peer observation).

Table 5 Definition of realist terms used in Work Package 3

Term	Definition
Context	Individual, team, organisational, or other factors that enable or constrain the operation of mechanisms.(40) This includes social phenomena such as rules, norms and values, meaning that contexts are not straightforwardly analogous with settings.(41)
Mechanism	The interaction of a programme's resources or opportunities with individuals' or teams' reasoning.(40)
Outcome	The 'demi-regular' occurrences arising from particular configurations of contexts and mechanisms.(42) Consistent with the recognition in realist ontology of the dynamic and non-linear nature of open systems in the social world,(43) 'outcomes' may be better understood as semi-stable processes.

For peer review only

Programme Theory	A middle-range theoretical explanation of how (implementation) programme activities relate to underlying theory. Even if not explicitly stated, programme theories contain ideas about how best to address challenges to achieving intended goals (including how to proactively manage these challenges)(44)

Identification, sampling and consent

Surveys

All hospice staff involved in direct patient care or management, as well as those directly involved in patient care (volunteers, support staff, board members with a hospice governance role) will be eligible. Eligible participants will be sent a link to the anonymous survey, for which completion online will be taken as implied consent.

Interviews

A purposive sampling strategy at each site will draw from a sampling frame that includes all healthcare assistants, nurses, allied health professionals, doctors, volunteers, care managers and board members at each study site. Within the constraints of an exploratory sample size (five staff and volunteers, and two members of management and/or executive board at each site), we shall endeavour to maximise variation in participant characteristics and roles, prioritising sampling that will enable comparison between those who do and do *not* take part. Informed consent will be obtained. Interviews will be conducted at a time suitable for participants and may be face-to-face or remote, according to participant preference.

Data collection and analysis

Staff and volunteers' pre- and post-implementation experiences (survey):

Survey using a modified and piloted Normalisation Measurement Instrument (NoMad).(45) of staff and volunteers' perceptions and experiences of implementation, in relation to each NPT mechanism, before and after using the CLECC-Pal implementation strategy.

Quantitative Likert scale responses will be analysed descriptively using radar plots. Free-text responses will be deductively thematically-analysed using the framework of NPT mechanisms (coherence, cognitive participation, collective action, and reflexive monitoring), allowing for inductive thematic analysis if responses do not fit within the framework. Thematic patterns and outliers will be identified. The analysis will also inform the structure, content, and focus of the staff and volunteer interviews.

Staff and volunteers' post-implementation experiences (interviews):

Realist interviews are distinct from conventional qualitative semi-structured interviews as they adopt a 'teacher-learner' approach in which theory is presented to participants so that they can communicate their own experiences and views that may refute, refine, or expand the theory.(46) In practice, the realist interviewer presents theory (context-mechanism-outcome configurations) in a form comprehensible to the participant and follows-up flexibly with further questions tailored to the participant's understanding, to ensure that the discussion enables theory-refinement rather than simply a discussion of experiences.

For peer review only

Interviews will build on Murray et al's.(47) operationalisation of NPT for the development and optimisation of interventions within trials (see Table 6).

Interview topics will include, but not be limited to, experiences of CLECC-Pal's acceptability and fit, rationale for any modifications to CLECC-Pal, perceived changes in communication between those caring for patients at-risk of delirium, changes in care practices, perceptions about how CLECC-Pal is achieving (or not) the intended effects and, if appropriate, how these impacts could be sustained. Interview questions will be informed by emerging site-specific data from the co-design and feasibility work packages, as well as from the process evaluation survey. Graphical summaries of data, such as radar plots, will be used in the interviews to communicate this emerging data to participants, link to theory, and to support discussion that enables implementation theory to be refined.(46, 48) Views of study processes will also be sought. It is envisaged that interviews will last no longer than 30 minutes, but participants will be given the opportunity for a longer interview if they wish.

Interviews will be recorded and transcribed. Before commencing analysis, interview transcripts will be read and re-read to allow familiarisation with the content that will enable theory-building and refinement rather than rote coding of contexts, mechanisms and outcomes (although coding of these configurations may also play an important role in theory-building and refinement). Analysis to identify contextualised explanations of how mechanisms of implementation are understood to lead to certain outcomes will be structured using the reasoning processes identified by Pawson (juxtaposition, reconciliation, adjudication, consolidation, and situating(49)). We shall operationalise these reasoning processes using the analytic questions for building and refining programme theory identified by Pearson et al.(50)

Work Package 3 methods and findings will be reported consistent with the RAMESES reporting standards.(42)

For peer review only

Table 6 Normalisation Process Theory 'Contribution' mechanisms and their relationship to data collection in interviews

Mechanism	Definition(36)	Theoretical propositions(37)	Potential interview questions(47)
1.Coherence	Agents attribute meaning to a complex intervention and make sense of its possibilities within their field of agency. They frame how participants make sense of, and specify, their involvement in a complex intervention.	1.1 Embedding is dependent on work that defines and organises a practice as a cognitive and behavioural ensemble.<br>1.2 Embedding work is shaped by factors that promote or inhibit actors' apprehension of a practice as meaningful.<br>1.3 The production and reproduction of coherence in a practice requires that actors collectively invest meaning in it.	Is CLECC-Pal:<br>- easy to describe?<br>- clearly distinct from other strategies?<br>- have a clear purpose for all participants?<br>Do participants have a shared sense of purpose?<br>What benefits will the intervention bring and to whom?<br>Are these benefits likely to be valued by potential participants?<br>Will CLECC-Pal fit with the overall goals and activity of the organisation?
2.Cognitive Participation	Agents legitimise and enrol themselves and others into a complex intervention. They frame how participants become members of a specific community of practice.	2.1 Embedding is dependent on work that defines and organises the actors implicated in a practice.<br>2.2 Embedding work is shaped by factors that promote or inhibit actors' participation.<br>2.3 The production and reproduction of a practice requires that actors collectively invest commitment in it.	Are target user groups likely to think that CLECC-Pal is a good idea?<br>Will they see the point of CLECC-Pal?
3.Collective Action	Agents mobilise skills and resources and enact a complex intervention. They frame how participants realise and perform the intervention in practice.	3.1 Embedding is dependent on work that defines and operationalises a practice.<br>3.2 Embedding work is shaped by factors that promote or inhibit actors' enacting it.<br>3.3 The production and reproduction of a practice requires that actors collectively invest effort in it.	How will CLECC-Pal affect the work of user groups?<br>Will CLECC-Pal promote or impede their work?<br>Will staff require extensive training before they can use CLECC-Pal?<br>How compatible with existing work practices is CLECC-Pal?<br>What impact will CLECC-Pal have on division of labour, resources, power, and responsibility between different professional groups?<br>Will CLECC-Pal fit with the overall goals and activity of the organisation?

Mechanism	Definition(36)	Theoretical propositions(37)	Potential interview questions(47)
4.Reflexive Monitoring	Agents assemble and appraise information about the effects of a complex intervention within their field of agency, and utilise that knowledge to reconfigure social relations and action. They frame how participants collect and utilise information about the effects of the intervention.	4.1 Embedding is dependent on work that defines and organises the everyday understanding of a practice.<br>4.2 Embedding work is shaped by factors that promote or inhibit appraisal.<br>4.2 The production and reproduction of a practice requires that actors collectively invest in its understanding.	How are users likely to perceive CLECC-Pal once it has been used for a while?<br>Is CLECC-Pal likely to be perceived as advantageous for patients or staff?<br>Will it be clear what effects CLECC-Pal has had?<br>Can users contribute feedback about CLECC-Pal once it is in use?<br>Can CLECC-Pal be adapted or improved on the basis of experience?

For peer review only

Ethical considerations

Ethical approval for the study has been obtained from Hull York Medical School Ethics Committee (Ref.: 21/23), Health Research Authority Research Ethics Committee Wales REC7 (Ref.: 21/WA/0180) and Health Research Authority Confidentiality Advisory Group (Ref.: 21/CAG/0071). Confidentiality Advisory Group approval allows the study researcher access to the clinical records to extract data without patient consent. The study is publicised in the hospices during the data collection period and patients/representatives may opt out if they do not wish their data to be used.

Progression to an evaluative study

In developing this protocol we have considered the balance between scientific rigour and practical considerations of a number of future evaluative study designs. For example an interrupted time series design would enable naturalistic data collection, but powering the study would likely require 12 months pre- and post-intervention data collection.(51) We also considered a randomised stepped wedge design, but considered implementation research permutations of this design unlikely to be feasible due to the real-world setting (if using a head-to-head rollout design) or length of time required (if using a pairwise enrolment rollout design).(52)

Consistent with current thinking in implementation research for investigators to consider quasi-experimental study designs that can assess the impact of context over time(53), we plan to work towards an evaluative study design that uses natural variation in the introduction of the implementation strategy to allow a *non-randomised* stepped wedge design (CLECC-Pal supported delirium care vs. delirium care as usual). Our audit data indicate that this would be realistic given an annual admission rate of 192-384 in the 10-20 bedded study site hospices which have a 40-60% incidence of delirium.

Whilst hospices are relatively homogeneous in terms of care delivery by health professionals (e.g. standardised national training programme for doctors, national standards for nursing practice), the wide referral base of hospices mean that in-patients tend to be heterogeneous in relation to type and stage of disease, ethnicity, socio-economic status, and so on. For the future evaluative study, we shall estimate the intraclass correlation coefficient (ICC) using pre-intervention patient outcome data (delirium-free days) from the feasibility study, thus enabling a sample size calculation powered on the primary outcome for the future evaluative study.

We are mindful of a recent systematic review of feasibility studies which identified a lack of consistency in the use of terminology, a predominance of feasibility issues relating to preparation for randomised-controlled trials, and an absence of clear guidance about when "sufficient insight about uncertainties" had been achieved for progression to an evaluation study.(54, p.10) However, we are confident in stating minimum recruitment targets for the use of CLECC-Pal (fidelity to core components) and 4AT screening tool at baseline and daily, that will be necessary for a future evaluative study to be considered feasible:

- ≥80%, proceed
- 60 – 80% with mitigating factors, proceed
- <60% not feasible

Dissemination

The primary objective of this study is to inform a future quasi-experimental multi-site comparative evaluation by demonstrating the feasibility of the implementation strategy ('intervention'), participant recruitment, and data collection, as well as informing decision-making about the most appropriate study design for a future multi-site comparative evaluation. However, as argued by Thabane et al.,(55) communicating findings from feasibility studies remains critically important for ensuring that resources are not spent on either duplicating the feasibility study or funding research uninformed by the findings of a relevant feasibility study.

A full report of the study's methods and findings will be prepared for the funder and a manuscript reporting the findings submitted to an open access peer-reviewed journal. The study's findings will be submitted for oral presentation at one national health services research conference and one international palliative care conference. A Plain English summary of study findings will be prepared for distribution through palliative care clinical networks (including Hospice UK) and Public Involvement groups.

Author contributions: MP and MJ led study conceptualisation and design, with contributions from CJ, JB and NS. MP and MJ led development of analysis plans, with contributions from CJ, CH and MT. MP led the writing process and drafted the original protocol with input from GJ and MJ. Critical review of the protocol and contributions to refinement to: co-design work package from GJ, MO, IF and MT; feasibility work package from GJ, CJ, JB, IF, CH, MO, KS, NS, MT and MJ; process evaluation work package from GJ, CJ, IF, MT and MJ. All authors take responsibility for the protocol and approved the final version of this paper.

Funding statement: This work is supported by Yorkshire Cancer Research (Award reference number HEND405DEL).

Competing interests statement: The authors declared no potential conflicts of interest with respect to the research, authorship, and/or publication of this article.

Acknowledgements: Creating Learning Environments for Compassionate Care (CLECC) © 2020 University of Southampton. Used under non-exclusive licence.

Figures:

Figure 1 Study flowchart and timeline summary

References:

1. American Psychiatric Association. Diagnostic and Statistical Manual of Mental Disorders, Fifth Edition (DSM-5). Arlington, VA: American Psychiatric Publisher; 2013.
2. Hosie A, Davidson PM, Agar M, Sanderson CR, Phillips J. Delirium prevalence, incidence, and implications for screening in specialist palliative care inpatient settings: A systematic review. Palliative Medicine. 2012;27:486–98.
3. Fang CK, Chen HW, Liu SI, Lin CJ, Tsai LY, Lai YL. Prevalence, detection and treatment of delirium in terminal cancer inpatients: a prospective survey. Jpn J Clin Oncol. 2008;38(1):56-63.
4. Lawlor PG, Gagnon B, Mancini IL, Pereira JL, Hanson J, Suarez-Almazor ME, et al. Occurrence, causes, and outcome of delirium in patient with advanced cancer: a prospective study. Arch Intern Med. 2000;160(6):786-94.

For peer review only

5. Weinrebe W, Johannsdottir E, Karaman M, Fusgen I. What does delirium cost? An economic evaluation of hyperactive delirium. Z Gerontol Geriatr. Z Gerontol Geriatr. 2016;49(1):52-8.
6. Zaubler TS, Murphy K, Rizzuto L, Santos R, Skotzko C, Giordano J, et al. Quality improvement and cost savings with multicomponent delirium interventions: replication of the Hospital Elder Life Program in a community hospital. Psychosomatics. 2013;54(3):219-26.
7. Watt CL, Momoli F, Ansari MT, Sikora L, Bush SH, Hosie A, et al. The incidence and prevalence of delirium across palliative care settings: A systematic review. Palliative Medicine. 2019;DOI: 10.1177/0269216319854944.
8. Oh ES, Fong TG, Hshieh TT, Inouye SK. Delirium in older persons: Advances in diagnosis and treatment. JAMA. 2017;318(12):1161-74.
9. Thom RP, Levy-Carrick NC, Bui M, Silbersweig D. Delirium. American Journal of Psychiatry. 2019;176(10):785-93.
10. Leslie DL, Inouye SK. The importance of delirium: economic and societal costs. J Am Geriatr Soc. 2011;59 (Suppl 2):S241-3.
11. NICE. Delirium: prevention, diagnosis and management - Clinical Guideline 103. London: National Institute for Health & Care Excellence; 2010.
12. Scottish Intercollegiate Guidelines Network. Risk reduction and management of delirium: A national clinical guideline. Edinburgh: NHS Scotland; 2019.
13. Australian Commission on Safety and Quality in Health Care. Delirium Clinical Care Standard. Sydney: ACSQHC; 2016.
14. Featherstone I, Hosie A, Siddiqi N, Grassau P, Bush SH, Taylor J, et al. The experience of delirium in palliative care settings for patients, family, clinicians and volunteers: A qualitative systematic review and thematic synthesis. Palliative Medicine. 2021;35(6):988-1004.
15. Boland JW, Kabir M, Bush SH, Spiller JA, Johnson MJ, Agar M, et al. Delirium management by palliative medicine specialists: a survey from the association for palliative medicine of Great Britain and Ireland. BMJ Supportive & Palliative Care. 2019.
16. Woodhouse R, Siddiqi N, Boland JW, Featherstone I, Johnson MJ. Screening for delirium: A survey of delirium screening practice in specialist palliative care units in the UK. BMj Supportive & Palliative Care. 2020;[Submitted for publication].
17. Steinhauser KE, Christakis NA, Clipp EC. Factors Considered Important at the End of Life by Patients, Family, Physicians, and Other Care Providers. JAMA. 2000;284(19):2476-82.
18. Brajtman S, Higuchi K, McPherson C. Caring for patients with terminal delirium: palliative care unit and home care nurses' experiences. International Journal of Palliative Nursing. 2006;12(4):150-6.
19. Hosie A, Lobb E, Agar M, Davidson PM, Phillips J. Identifying the barriers and enablers to palliative care nurses' recognition and assessment of delirium symptoms: a qualitative study. Journal of Pain & Symptom Management. 2014;48(5):815-30.
20. Bridges J, Pickering RM, Barker H, Chable R, Fuller A, Gould L, et al. Implementing the Creating Learning Environments for Compassionate Care (CLECC) programme in acute hospital settings: a pilot RCT and feasibility study. Health Serv Deliv Res. 2018;6(33).
21. Bridges J, Gould L, Libberton P, Schoonhoven L. Creating learning environments for compassionate care: a programme to support nursing leaders and teams to deliver compassionate hospital care - Manual for Practice Development Leads. Southampton: University of Southampton; 2020.
22. Hoffmann TC, Glasziou PP, Boutron I, Milne R, Perera R, Moher D, et al. Better reporting of interventions: template for intervention description and replication (TIDieR) checklist and guide. BMJ. 2014;348:g1687.
23. UK Public Involvement Standards Development Partnership. UK Standards for Public Involvement. London: NIHR Central Commissioning Facility; 2019.
24. Bate P, Robert G. Experience-based design: from redesigning the system around the patient to co-designing services with the patient. Qual Saf Health Care. 2006;15(5):307-10.
25. Locock L, Robert G, Boaz A, Vougioukalou S, Shuldham C, Fielden J, et al. Testing accelerated experience-based co-design: a qualitative study of using a national archive of patient experience narrative interviews to promote rapid patient-centred service improvement. Health Serv Deliv Res. 2014;2(4).
26. Pearson N, Naylor P-J, Ashe MC, Fernandez M, Yoong SL, Wolfenden L. Guidance for conducting feasibility and pilot studies for implementation trials. Pilot and Feasibility Studies. 2020;6(167).
27. Iedema R, Merrick E, Piper D, Britton K, Gray J, Verma R, et al. Codesigning as a discursive practice in emergency health services: the architecture of deliberation. Journal of Applied Behavioral Science. 2010;46(1):73-91.

For peer review only

28. INVOLVE. Guidance on co-producing a research project. Southampton: INVOLVE; 2018.
29. Inouye SK, Leo-Summers L, Zhang Y, Bogardus ST, Leslie DL, Agostini JV. A chart-based method for identification of delirium: validation compared with interviewer ratings using the Confusion Assessment Method. J Am Geriatr Soc. 2005;53:312-8.
30. Ritchie J, Lewis J, McNaughton-Nicholls C, Ormston R, editors. Qualitative Research Practice: A guide for social science students and researchers. 2nd edition ed. London: Sage; 2013.
31. Moore GF, Audrey S, Barker M, Bond L, Bonell C, Hardeman W, et al. Process evaluation of complex interventions: Medical Research Council guidance. BMJ. 2015;350:h1258.
32. Murdoch J. Process evaluation for complex interventions in health services research: analysing context, text trajectories and disruptions. BMC Health Serv Res. 2016;16(407).
33. Wells M, Williams B, Treweek S, Coyle J, Taylor J. Intervention description is not enough: evidence from an in-depth multiple case study on the untold role and impact of context in randomised controlled trials of seven complex interventions. Trials. 2012;13:95-111.
34. Pawson R. The Science of Evaluation: A Realist Manifesto. London: Sage; 2013.
35. O'Brien R, Buston K, Wight D, McGee E, White J, Henderson M. A realist process evaluation of Enhanced Triple P for Baby and Mellow Bumps, within a Trial of Healthy Relationship Initiatives for the Very Early years (THRIVE): study protocol for a randomized controlled trial. Trials. 2019;20(1):351.
36. May C. Towards a general theory of implementation. Implementation science : IS. 2013;8:18.
37. May C, Finch T. Implementing, Embedding, and Integrating Practices: An Outline of Normalization Process Theory. Sociology. 2009;43(3):535-54.
38. May CR, Cummings A, Girling M, Bracher M, Mair FS, May CM, et al. Using Normalization Process Theory in feasibility studies and process evaluations of complex healthcare interventions: a systematic review. Implementation science : IS. 2018;13(1):80.
39. Hawe P, Shiell A, Riley T. Theorising interventions as events in systems. American journal of community psychology. 2009;43(3-4):267-76.
40. Pearson M, Chilton R, Wyatt K, Abraham C, Ford T, Woods HB, et al. Implementing health promotion programmes in schools: a realist systematic review of research and experience in the United Kingdom. Implementation science : IS. 2015;10(1):149.
41. Pawson R, Tilley N. Realistic Evaluation. London: Sage Publications; 1997.
42. Wong G, Westhorp G, Manzano A, Greenhalgh J, Jagosh J, Greenhalgh T. RAMESES II reporting standards for realist evaluations. BMC medicine. 2016;14(1):96.
43. Sayer A. Realism and Social Science. London: Sage; 2000.
44. Pawson R, Owen L, Wong G. The Today Programme's contribution to Evidence-Based Policy. Evaluation. 2010;16(2):211-3.
45. Finch TL, Girling M, May CR, Mair FS, Murray E, Treweek S, et al. Nomad: Implementation measure based on Normalization Process Theory. [Measurement instrument]. 2015 [01/12/2021]. Available from: http://www.normalizationprocess.org.
46. Manzano A. The craft of interviewing in realist evaluation. Evaluation. 2016;22(3):342-60.
47. Murray E, Treweek S, Pope C, MacFarlane A, Ballini L, Dowrick C, et al. Normalisation process theory: a framework for developing, evaluating and implementing complex interventions. BMC Medicine. 2010;8(63).
48. Mukumbang FC, Marchal B, Van Belle S, van Wyk B. Using the realist interview approach to maintain theoretical awareness in realist studies. Qualitative Research. 2019:146879411988198.
49. Pawson R. Evidence-Based Policy: A Realist Perspective. London: Sage Publications; 2006.
50. Pearson M, Brand SL, Quinn C, Shaw J, Maguire M, Michie S, et al. Using realist review to inform intervention development: methodological illustration and conceptual platform for collaborative care in offender mental health. Implementation science : IS. 2015;10(1):134.
51. Zhang F, Wagner AK, Ross-Degnan D. Simulation-based power calculation for designing interrupted time series analyses of health policy interventions. Journal of Clinical Epidemiology. 2011;64:1252-61.
52. Brown CH, Curran G, Palinkas LA, Aarons GA, Wells KB, Jones L, et al. An Overview of Research and Evaluation Designs for Dissemination and Implementation. Annu Rev Public Health. 2017;38:1-22.
53. Kemp CG, Wagenaar BH, Haroz EE. Expanding Hybrid Studies for Implementation Research: Intervention, Implementation Strategy, and Context. Frontiers in Public Health. 2019;7.
54. Hallingberg B, Turley R, Segrott J, Wight D, Craig P, Moore L, et al. Exploratory studies to decide whether and how to proceed with full-scale evaluations of public health interventions: a systematic review of guidance. Pilot and Feasibility Studies. 2018;4(104).

55. Thabane L, Ma J, Chu R, Cheng J, Ismaila A, Rios LP, et al. A tutorial on pilot studies: the what, why and how. BMC Medical Research Methodology. 2010;10(1).

For peer review only

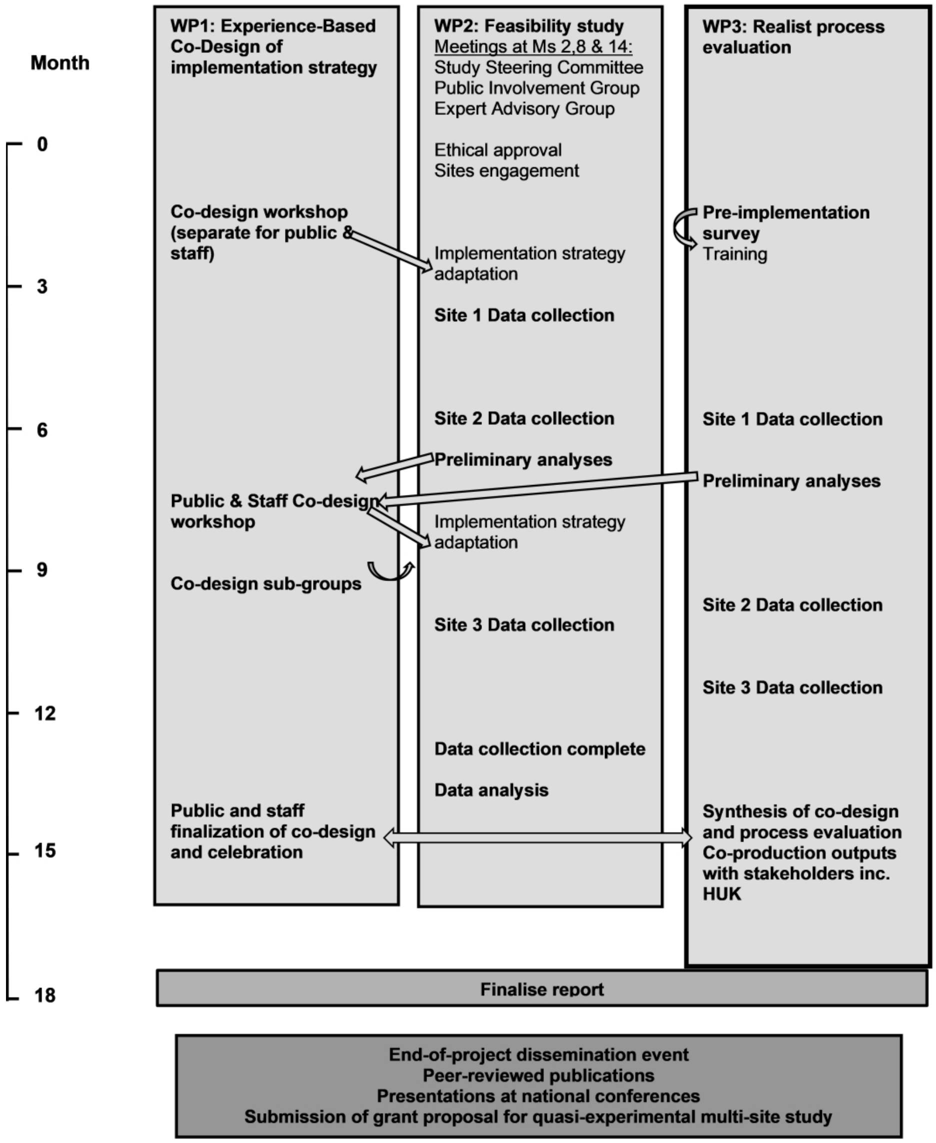

Figure 1 Study flowchart and timeline summary

1034x1326mm (96 x 96 DPI)

Online supplemental file 1

'Improving the Detection, Assessment, Management, and Prevention of Delirium in Hospices (the DAMPen-D study): Protocol for a co-design and feasibility study of a flexible and scalable implementation strategy to deliver guideline-adherent delirium care.'

**Case Number_Pre**	Non-identifiable ID number	N/A	Alphnumeric
**Age_Pre**	Patient age	Integer	Years
**Sex_Pre**	Patient gender	Categorical	Male, female, other
**Diagnosis_Pre**	Patient diagnosis	String	Cancer etc.
**Ethnicity_pre**	Patient ethnicity	Categorical	Black, white etc.
**IMD score_Pre**	Postcode converted	Float	
**Adm_Ac_conf_state_Pre**	Evidence of acute confusional state on admission	Binary	Yes/No
**Adm_screen_Pre**	Patient screened for delirium on admission	Binary	Yes/No
**Adm_Screen_by_Pre**	If screened, who completed screening	Categorical	Doctor, Nurse practitioner, Registered Nurse, Other (specify)
**Adm_screen_type_Pre**	If screened, name of screening tool	Alphanumeri c	4AT etc.
**Adm_screen_result_Pre**	If screened, result	Binary	Positive/Negative
**Adm_no_screen_just-Pre**	Was justification given for not screening	Binary	Yes/No
**Adm_no-screen_just-verbatim-Pre**	Was justification given for not screening	String	Verbatim text

Online supplemental file 1
'Improving the Detection, Assessment, Management, and Prevention of Delirium in Hospices (the DAMPen-D study): Protocol for a co-design and feasibility study of a flexible and scalable implementation strategy to deliver guideline-adherent delirium care.'

**Adm_risk-ass-Pre**	If screening negative or not done was risk assessment carried out?	Binary	Yes/No
**Adm-risk-ass_result_Pre**	If risk assessment carried completed results	Binary	Positive/Negative
**Adm_prev_meas_Pre**	If risk assessment positive were preventive measures put in place	Binary	Yes/No
**Adm_Judge_rationale_pre**	If researcher judgement was required for any of above, give rationale	String	Free text
**Dur_adm_Ac_conf_state_pre**	Evidence of acute confusional state during admission	Binary	Yes/No
**Dur_adm_ Multi_ep_cog_dys_Pre**	Multiple episodes of cognitive dysfunction during admission	Binary	Yes/No
**Dur_adm_ Multi_ep_cog_dys_no_Pre**	Multiple episodes of cognitive dysfunction during admission	Integer	1,2,3

For peer review only - http://bmjopen.bmj.com/site/about/guidelines.xhtml

Online supplemental file 1
'Improving the Detection, Assessment, Management, and Prevention of Delirium in Hospices (the DAMPen-D study): Protocol for a co-design and feasibility study of a flexible and scalable implementation strategy to deliver guideline-adherent delirium care.'

**Dur_adm_screen_Pre**	If patient had multiple episodes was the patient screened	Binary	Yes/No
**Dur_adm_screen_type_Pre**	If screened, name of tool	Alphanumeri c	4AT etc.
**Dur_Adm_Screen_result_Pre**	Result of screening during admission	Binary	Positive/negative
**Dur_adm_Sceen_by_Pre**	Who completed screening during admission	Categorical	Doctor, Nurse practitioner, Registered Nurse, Other (specify)
**Dur_adm_Judge_rationale_Pre**	If researcher judgement was required for any of above, give rationale	String	Verbatim text
**Case_rec_ac_conf_reported_by_Pr e**	Who reported the first episode of acute confusion in the case record	Categorical	Doctor, Nurse practitioner, Registered Nurse, Other (specify)
**Case_rec_date_first_ep_Pre**	Date of first episode of acute confusion in the case record	Date	10.10.2021
**Case_rec_time_first_ep_Pre**	Time of first episode of acute confusion in case record	Time	24hr format

Online supplemental file 1
'Improving the Detection, Assessment, Management, and Prevention of Delirium in Hospices (the DAMPen-D study): Protocol for a co-design and feasibility study of a flexible and scalable implementation strategy to deliver guideline-adherent delirium care.'

**Case_rec_verbatim_ref_ac_conf_Pre**	Describe each reference to acute confusion in the case record	String	Verbatim text
**Case_rec_ac_conf_tot_days_Pre**	Total duration of acute confusion in days as determined by all the references in the case record	Integer	5 (days) or 0 days if none
**Case_rec_Improve_revers_Pre**	Any evidence of improvement or reversibility of acute confusion during the stay	Categorical	Yes/No/Unsure
**Case_rec_ev_descr_pre**	Describe evidence of reversibility	String	Free text
**Case_rec_Del_present_Pre**	Delirium present	Categorical	Yes/No
**Case_rec_subtype_Pre**	If delirium present what subtype	Categorical	Hypo/Hyper/Mixed
**Case_rec_del_med_ass_Pre**	Medical assessment (DSM-V delirium assessment) to assess for delirium	Binary	Yes/No
**Case_rec_diag_doc_Pre**	Diagnosis of delirium recorded	Categorical	Yes/No

For peer review only - http://bmjopen.bmj.com/site/about/guidelines.xhtml

Online supplemental file 1
'Improving the Detection, Assessment, Management, and Prevention of Delirium in Hospices (the DAMPen-D study): Protocol for a co-design and feasibility study of a flexible and scalable implementation strategy to deliver guideline-adherent delirium care.'

**Case_rec_judge_rationale_Pre**	If researcher judgement was required for any of above, give rationale	String	Free text
**Invest_del_ ass_rev_cause_Pre**	Assessment for reversible causes of delirium	Binary	Yes/No
**Invest_med_rev_Pre**	Was a medication review conducted	Binary	Yes/No
**Invest_rev_cause_treat_Pre**	Was a treatment instigated for a reversible cause of delirium	Binary	Yes/No
**Invest_ judge_rationale_Pre**	If researcher judgement was required for any of above, give rationale	String	Free text
**Del_care_plan_Pre**	Delirium care plan documented	Binary	Yes/no
**Del_sev_Pre**	Was delirium severity assessed	Categorical	RASS-PAL + hallucination,RASS-PAL only, hallucination only, other specify, No

For peer review only - http://bmjopen.bmj.com/site/about/guidelines.xhtml

Online supplemental file 1
'Improving the Detection, Assessment, Management, and Prevention of Delirium in Hospices (the DAMPen-D study): Protocol for a co-design and feasibility study of a flexible and scalable implementation strategy to deliver guideline-adherent delirium care.'

**Harm_distress_behaviour_Pre**	Did patient display behaviours harmful or distressing to self or others	Binary	Yes/No
**Sedative_admin_during_del_Pre**	Was sedative administered during period of delirium	Binary	Yes/No
**Sedative_med_type**	Sedative medication type	String	Name of medication
**Sed_ind_Pre**	Sedative medication administered for	Categorical	Delirium, anxiety, breathlessness, nausea, terminal agitation, other, unclear
**Del_risk_discuss_patient_fam**	Was delirium risk and prevention discussed with patients and families of patients without delirium on admission	Categorical	Yes/No/unable
**Del_ep_discuss_patient_Pre**	Was episode of delirium discussed with the patient	Categorical	Yes/No/Unable

For peer review only - http://bmjopen.bmj.com/site/about/guidelines.xhtml

Online supplemental file 1
'Improving the Detection, Assessment, Management, and Prevention of Delirium in Hospices (the DAMPen-D study): Protocol for a co-design and feasibility study of a flexible and scalable implementation strategy to deliver guideline-adherent delirium care.'

**Del_ep_discuss_patient_family_Pre**	Was episode of delirium discussed with the patient's family	Categorical	Yes/No/Unable
**Del_info_Pre**	Was any written information about delirium provided to patient or family	Categorical	Yes/No/Unable

For peer review only - http://bmjopen.bmj.com/site/about/guidelines.xhtml

CONSORT 2010 checklist of information to include when reporting a pilot or feasibility trial*

Section/Topic	Item No	Checklist item	Reported on page No
**Title and abstract**			
	1a	Identification as a pilot or feasibility randomised trial in the title	1
	1b	Structured summary of pilot trial design, methods, results, and conclusions (for specific guidance see CONSORT abstract extension for pilot trials)	2
**Introduction**			
Background and objectives	2a	Scientific background and explanation of rationale for future definitive trial, and reasons for randomised pilot trial	3-4
	2b	Specific objectives or research questions for pilot trial	4, 6
**Methods**			
Trial design	3a	Description of pilot trial design (such as parallel, factorial) including allocation ratio	NA (protocol)
	3b	Important changes to methods after pilot trial commencement (such as eligibility criteria), with reasons	NA (protocol)
Participants	4a	Eligibility criteria for participants	9
	4b	Settings and locations where the data were collected	4
	4c	How participants were identified and consented	9, 12, 15
Interventions	5	The interventions for each group with sufficient details to allow replication, including how and when they were actually administered	5
Outcomes	6a	Completely defined prespecified assessments or measurements to address each pilot trial objective specified in 2b, including how and when they were assessed	9-11, 12
	6b	Any changes to pilot trial assessments or measurements after the pilot trial commenced, with reasons	NA (protocol)
	6c	If applicable, prespecified criteria used to judge whether, or how, to proceed with future definitive trial	16
Sample size	7a	Rationale for numbers in the pilot trial	9-10
	7b	When applicable, explanation of any interim analyses and stopping guidelines	NA
Randomisation:			
Sequence generation	8a	Method used to generate the random allocation sequence	NA
	8b	Type of randomisation(s); details of any restriction (such as blocking and block size)	NA
Allocation concealment mechanism	9	Mechanism used to implement the random allocation sequence (such as sequentially numbered containers), describing any steps taken to conceal the sequence until interventions were assigned	NA

For peer review only

Implementation	10	Who generated the random allocation sequence, who enrolled participants, and who assigned participants to interventions	NA
Blinding	11a	If done, who was blinded after assignment to interventions (for example, participants, care providers, those assessing outcomes) and how	NA
	11b	If relevant, description of the similarity of interventions	NA
Statistical methods	12	Methods used to address each pilot trial objective whether qualitative or quantitative	10, 12
**Results**			
Participant flow (a diagram is strongly recommended)	13a	For each group, the numbers of participants who were approached and/or assessed for eligibility, randomly assigned, received intended treatment, and were assessed for each objective	NA (protocol)
	13b	For each group, losses and exclusions after randomisation, together with reasons	NA (protocol)
Recruitment	14a	Dates defining the periods of recruitment and follow-up	NA (protocol)
	14b	Why the pilot trial ended or was stopped	NA (protocol)
Baseline data	15	A table showing baseline demographic and clinical characteristics for each group	NA (protocol)
Numbers analysed	16	For each objective, number of participants (denominator) included in each analysis. If relevant, these numbers should be by randomised group	NA (protocol)
Outcomes and estimation	17	For each objective, results including expressions of uncertainty (such as 95% confidence interval) for any estimates. If relevant, these results should be by randomised group	NA (protocol)
Ancillary analyses	18	Results of any other analyses performed that could be used to inform the future definitive trial	NA (protocol)
Harms	19	All important harms or unintended effects in each group (for specific guidance see CONSORT for harms)	NA (protocol)
	19a	If relevant, other important unintended consequences	NA (protocol)
**Discussion**			
Limitations	20	Pilot trial limitations, addressing sources of potential bias and remaining uncertainty about feasibility	NA (protocol)
Generalisability	21	Generalisability (applicability) of pilot trial methods and findings to future definitive trial and other studies	16
Interpretation	22	Interpretation consistent with pilot trial objectives and findings, balancing potential benefits and harms, and considering other relevant evidence	NA (protocol)
	22a	Implications for progression from pilot to future definitive trial, including any proposed amendments	16
**Other information**			
Registration	23	Registration number for pilot trial and name of trial registry	2
Protocol	24	Where the pilot trial protocol can be accessed, if available	NA (protocol)
Funding	25	Sources of funding and other support (such as supply of drugs), role of funders	17
	26	Ethical approval or approval by research review committee, confirmed with reference number	16

Citation: Eldridge SM, Chan CL, Campbell MJ, Bond CM, Hopewell S, Thabane L, et al. CONSORT 2010 statement: extension to randomised pilot and feasibility trials. BMJ. 2016;355.
*We strongly recommend reading this statement in conjunction with the CONSORT 2010, extension to randomised pilot and feasibility trials, Explanation and Elaboration for important clarifications on all the items. If relevant, we also recommend reading CONSORT extensions for cluster randomised trials, non-inferiority and equivalence trials, non-pharmacological treatments, herbal interventions, and pragmatic trials. Additional extensions are forthcoming: for those and for up to date references relevant to this checklist, see www.consort-statement.org.

For peer review only

BMJ Open

Improving the Detection, Assessment, Management, and Prevention of Delirium in Hospices (the DAMPen-D study): protocol for a co-design and feasibility study of a flexible and scalable implementation strategy to deliver guideline-adherent delirium care.

Journal:	*BMJ Open*
Manuscript ID	bmjopen-2021-060450.R1
Article Type:	Protocol
Date Submitted by the Author:	18-Mar-2022
Complete List of Authors:	Pearson, Mark; Hull York Medical School, Wolfson Palliative Care Research Centre<br>Jackson, Gillian; Hull York Medical School, Wolfson Palliative Care Research Centre<br>Jackson, Catriona; St James's University Hospital<br>Boland, Jason; Hull York Medical School,<br>Featherstone, Imogen; University of York, Department of Health Sciences<br>Huang, Chao; University of Hull<br>Ogden , Margaret; University of Stirling, Faculty of Social Sciences<br>Sartain, Kathryn; University of Hull; York and Scarborough Teaching Hospitals NHS Foundation Trust<br>Siddiqi, Najma; University of York, Psychiatry, Hull York Medical School, York and Bradford District Care NHS Foundation Trust, Bradford, UK; Research<br>Twiddy, Maureen; University of Hull<br>Johnson, Miriam; The University of Hull, Hull York Medical School
**Primary Subject Heading**:	Palliative care
Secondary Subject Heading:	Evidence based practice, Geriatric medicine
Keywords:	PALLIATIVE CARE, Delirium & cognitive disorders < PSYCHIATRY, Protocols & guidelines < HEALTH SERVICES ADMINISTRATION & MANAGEMENT

SCHOLARONE™
Manuscripts

Improving the Detection, Assessment, Management, and Prevention of Delirium in Hospices (the DAMPen-D study):

Protocol for a co-design and feasibility study of a flexible and scalable implementation strategy to deliver guideline-adherent delirium care.

Pearson, M.*[1], Jackson, G.[1], Jackson, C.[1,2], Boland, J.[1], Featherstone, I.[3], Huang, C.[4], Ogden, M.[5], Sartain, K.[6], Siddiqi, N.[3], Twiddy, M.[4] & Johnson, M.J[1]

* corresponding author: Dr Mark Pearson, Wolfson Palliative Care Research Centre, Hull York Medical School, Allam Medical Building, University of Hull, Hull, HU6 7RX, UK. Tel.: +44 1482 463335

[1] Wolfson Palliative Care Research Centre, Hull York Medical School, University of Hull, Hull, HU6 7RX, UK

[2] Leeds Teaching Hospitals NHS Trust, St James's University Hospital, Leeds, LS9 7TF, UK

[3] Department of Health Sciences, University of York, York, YO10 5DD, UK

[4] Institute of Clinical & Applied Health Research, Hull York Medical School, University of Hull, Hull, HU6 7RX, UK

[5] Public involvement member

[6] York and Scarborough Teaching Hospitals NHS Foundation Trust, York Hospital, Wigginton Road, York, YO31 8HE, UK

Author email addresses:
Pearson, M. mark.pearson@hyms.ac.uk
Jackson, G. gillian.jackson@hyms.ac.uk
Jackson, C. catriona.jackson@doctors.org.uk
Boland, J. jason.boland@hyms.ac.uk
Featherstone, I. imogen.featherstone@york.ac.uk
Huang, C. chao.huang@hyms.ac.uk
Ogden, M. margaretogden@hotmail.com
Sartain, K. kathryn.sartain@york.nhs.uk
Siddiqi, N. najma.siddiqi@york.ac.uk
Twiddy, M. maureen.twiddy@hyms.ac.uk
Johnson, M.J miriam.johnson@hyms.ac.uk

Word count (excluding title page, abstract, references, figures and tables): 4008

For peer review only

Abstract

Introduction Delirium is a complex condition in which altered mental state and cognition causes severe distress and poor clinical outcomes for patients and families, anxiety and stress for the health professionals and support staff providing care, and higher care costs. Hospice patients are at high risk of developing delirium, but there is significant variation in care delivery. The primary objective of this study is to demonstrate the feasibility of an implementation strategy (designed to help deliver good practice delirium guidelines), participant recruitment, and data collection.

Methods and analysis Three work packages in three hospices in the United Kingdom with public involvement in co-design, study management and stakeholder groups: 1) Experience-Based Co-Design to adapt an existing theoretically-informed implementation strategy (Creating Learning Environments for Compassionate Care (CLECC)) to implement delirium guidelines in hospices. 2) Feasibility study to explore ability to collect demographic, diagnostic, and delirium management data from clinical records (n=300), explanatory process data (number of staff engaged in CLECC activities, and reasons for non-engagement),and cost data (staff and volunteer hours and pay-grades engaged in implementation activities). 3) Realist Process Evaluation to assess the acceptability and flexibility of the implementation strategy (pre- and post-implementation surveys with hospice staff and management,n=30 at each time-point; interviews with hospice staff and management,n=15). Descriptive statistics, rapid thematic analysis, and a realist logic of analysis will be used be used to analyse quantitative and qualitative data, as appropriate.

Ethics and dissemination Ethical approval obtained: Hull York Medical School Ethics Committee (Ref.:21/23), Health Research Authority Research Ethics Committee Wales REC7 (Ref.:21/WA/0180) and Health Research Authority Confidentiality Advisory Group (Ref.:21/CAG/0071). Written informed consent will be obtained from interview participants. A results paper will be submitted to an open access peer-reviewed journal and a lay summary shared with study site staff and stakeholders.

Study registration: ISRCTN 55416525

Keywords: Delirium; palliative care; guideline implementation; co-design; feasibility; realist process evaluation

Article summary - Strength and limitations of this study:

- Innovative collaborative adaptation of a theoretically-informed implementation strategy (CLECC) to deliver guideline-adherent delirium care in hospices (CLECC-Pal), including evaluation of feasibility and acceptability of an implementation strategy before testing at scale.
- Research waste minimised and patient/carer burden eliminated through use of existing patient outcome and process data.
- Involvement of public members since study inception and throughout study delivery and management.
- Whilst the study hospices have diverse characteristics (locations, level of socio-economic deprivation, forms of governance), they are all drawn from a single region of the United Kingdom.
- The sample size for surveys and interviews may limit the extent to which the complexity of staff and management characteristics, views and experiences can be explored.

For peer review only

INTRODUCTION

Delirium is a complex condition characterised by fluctuating impairment of awareness, attention, and cognition.(1) Delirium causes severe distress for patients and families(2), anxiety and stress for the health professionals and support staff providing care,(3) poor clinical outcomes,(4, 5) and higher care costs (e.g. longer inpatient stays).(6, 7) People nearing the end of life have a high risk of delirium,(2) with risk factors such as medication, metabolic disturbance, pain, poor sleep, infection and dehydration acting cumulatively.(8) Effective delirium care is driven by prevention where possible, timely detection and non-pharmacological management, with pharmacological interventions if appropriate.(9, 10) Hospices are an important but under-researched setting for the prevention and management of delirium.

An international systematic review reported that one-third of people in adult palliative care settings had delirium on admission, with two-thirds developing delirium during the admission.(8) Across health services the health economic impact of delirium is significant. Although data are not available from palliative care settings, other estimates of health service costs from delirium show comparable costs to falls, diabetes and cardiovascular diseases.(11)

NICE Clinical Guideline 103(12) and other international guidelines(13) and standards,(14) recommend strategies for delirium assessment, prevention and management. However, this is difficult in practice, with a disconnection between improved levels of delirium knowledge and the capacity of palliative care practitioners to implement changes. A recent international qualitative systematic review identified that practical and emotional support were needed to enable staff to assess, prevent and manage delirium.(15)

A recent survey of palliative care doctors (n=335) in the United Kingdom found that 38% never used delirium guidelines and that only 13% of palliative care teams used a tool (rather than clinical judgement) to assess for delirium at first inpatient assessment, with even fewer (9%) using a tool on an ongoing basis.(16) Our survey of UK specialist palliative care units (n=220, mostly nurses)(17) found that only 10% ever used a delirium screening tool, with only 5% following NICE guidelines by screening on admission, and only 6% screening daily thereafter. The importance of delirium care has been recognised in a national survey of dying patients, with 92% rating ‘being mentally aware’ as “very important” and nearly as many (89%) citing ‘not being a burden on family’.(18)

Delirium detection, assessment, management and prevention is complex, depending on practical support (screening tools and clinical pathways) and communication (3, 19) between family and friends, volunteers, healthcare assistants (HCAs), nurses, allied health professionals (AHPs), social workers, doctors, hospice managers and board members. It also takes place at some of the most sensitive and emotionally-fraught times in the lives of patients and their families. Therefore, guideline implementation requires a relevant and flexible strategy based on an understanding of how adaptation for different settings can be attained whilst retaining effectiveness.

To address this gap in knowledge about how to implement guideline-adherent delirium care, we shall first adapt an existing theoretically-informed implementation strategy that has been tested in acute hospital wards (*Creating Learning Environments for Compassionate Care (CLECC)*). CLECC has been found to foster and legitimise the reflection, learning, mutual support and innovation that can enable team members to progress from knowing to doing.(20) It comprises a team study day, ward manager action learning sets, peer observations of practice, and involvement of all staff in mid-shift ‘cluster discussions’ and

For peer review only - http://bmjopen.bmj.com/site/about/guidelines.xhtml

twice-weekly reflective discussions,(21) and is shown mapped to the TIDieR checklist(22) in Table 1. We will then test the feasibility of a subsequent quasi-experimental study to evaluate the effect of the adapted CLECC (the intervention) on hospice staff delivery of guideline-adherent delirium care and subsequent improvement in patient outcomes (reduction in the number of delirium days).

Aims and objectives

This study will address key uncertainties about the implementation of guideline-adherent delirium care in hospices by demonstrating if it is possible to:

- Co-adapt an implementation strategy (Creating Learning Environments for Compassionate Care (CLECC)) for use in hospices (Work Package 1).
- Systematically and reliably collect data (including delirium diagnosis) from clinical records in a way that minimises burden for patients, families, and staff (Work Package 2).
- Collect measures of staff engagement with the implementation strategy, delivery of guideline-adherent delirium care, and the costs of staff involvement (Work Package 2).
- Collect explanatory process data about staff use of the implementation strategy (Work Package 3).
- Estimate the number of hospice sites and in-patient episodes needed for the planned national quasi-experimental study.

Work Package 1 commenced June 2021, with Work Packages 2 and 3 (and data collection) commencing August 2021. The study will be completed in February 2023.

METHODS AND ANALYSIS

Design summary

Table 2 presents the research questions and summarises the three Work Packages (WPs) that will enable the above aims and objectives to be met. Figure 1 shows the study timeline and how the work packages are inter-related.

Settings

Three adult hospices in northern England (United Kingdom). Two hospices in this study are located in socio-economically deprived urban areas (one with a significant minority ethnic group population) and one hospice in an affluent rural/urban area. One hospice is run by a national charity, with the other two hospices run by independent charities.

Patient and Public Involvement

This study supports the involvement of patient and public involvement (PPI) in accordance with the framework for good public involvement as detailed by the UK standards for public involvement.(23) Public involvement group members contributed to study design, with one member joining the monthly Study Management Group meetings, co-facilitating workshops (Work Package 1) and a further member Chairing the Study Steering Committee. The study's Public Involvement Group will meet three to four times over the duration of the study to discuss public involvement challenges in the research, the implications of emerging study

For peer review only

findings, and the development of public-facing research outputs and the next steps in the research cycle.

For peer review only

Table 1 CLECC(21) components mapped using TIDieR checklist(22)

Component	Why	What	Who	How	Where	When/How much	Tailoring & modifications	Fidelity
**Study day**	Prepare staff for the workplace elements of the intervention	**Procedure:** Introduction to CLECC Activities/discussion Questionnaires Film handouts **Materials:** PowerPoint presentation. Record of attendance. Summary of CLECC leaflet	Appointed hospice lead clinician	Classroom based to include all hospice staff	Comfortable classroom that is geographically separate from the workplace	One day at beginning of implementation period, but may require more than one study day to ensure maximum attendance	Pending Work Package 1 co-design workshops	Attendance and feedback data from hospice lead clinician.
**Action Learning sets**	Real problems from own practice and devise action plan to address	**Procedure:** Session 1: relationships & rules Session 2: valuing staff Session 3: enhancing capacity CLECC Session 4: influencing seniors	Experienced facilitator and 4-8 leads of comparable position	Face to face at hospice site	At hospice site	4 x4 hours action learning sets throughout intervention period	Pending Work Package 1 co-design workshops	Fidelity/ attendance
**Peer review**	Appreciate practice from observer perspective	**Procedure:** 2-3 x 1 hour observations Reflective summary **Materials:** Training video Poster of findings	2 team members nominate or nominated by lead and training given.	Outside of normal role to do this activity	At hospice site	Approximately 30 minute training video prior to commencing 2-3 x 1 hour observations throughout implementation	Pending Work Package 1 co-design workshops	Fidelity
**Mid-shift cluster discussions**	Opportunities for feedback, group problem solving and support to individual team members.	**Procedure:** Mid-shift 5 minute discussion	All team members on shift.	Mid-way through every shift.	At hospice site	5 minute discussion mid-shift, initially instigated by lead but then to be maintained by staff	Pending Work Package 1 co-design workshops	Fidelity
**Reflective discussions**	To prompt personal reflections and narratives about individual experiences	**Procedure:** Scheduled meetings or drop in sessions with planned activities **Materials:** Devise a sustainability plan	All team members, including senior staff and temporary staff.	Can be scheduled time during shift or drop-in sessions.	At hospice site, in a comfortable room on or near place of care.	Number of sessions dependent on the number of subjects needed to be discussed	Pending Work Package 1 co-design workshops	Fidelity

For peer review only

Table 2 Overview of study design

Work Package objective	Research question	Study type	Data collection	Timepoints
1. Refine CLECC-Pal implementation strategy	What are the core and adaptable components of an implementation strategy for guideline-adherent delirium care in hospices?	Experience-based co-design	Workshops	Before and during implementation
2. Demonstrate feasibility of future quasi-experimental study	Is it feasible to collect sufficient outcome data (both implementation and clinical), explanatory process data, and cost data in a future effectiveness evaluative study in palliative care settings?	Feasibility study	Patient demographics and delirium diagnosis & management (clinical records)<br><br>Number of staff engaged in CLECC-Pal	Baseline & follow-up<br><br>During implementation & follow-up
3. Assess acceptability and flexibility of CLECC-Pal implementation strategy	How can a co-designed implementation strategy for guideline-adherent delirium care be operationalised with fidelity to function in different hospice inpatient settings?	Realist process evaluation	Survey<br><br>Fidelity to CLECC-Pal<br><br>Interviews	Baseline & follow-up<br><br>Start, middle & end of 3-month period using CLECC-Pal<br><br>Follow-up

For peer review only

[Insert Figure 1 about here]

Work Package 1: Adaptation (Co-Design) of CLECC for guideline-adherent delirium care

An experience-based co-design (EBCD) group(24-26) of people with lived experience of delirium (themselves or in a family member or friend), staff and management from across the study sites and the region will meet for online workshops (maximum three hours duration) at months 2, 8, and 14 to adapt the CLECC strategy for use in hospices (see Figure 1). The first of these co-design workshops will be held separately for public and staff to facilitate reflection within a broader public or staff 'group' and to underpin interactions between public and staff at subsequent joint workshops. The interactions in these joint co-design workshops are considered essential for participants to share their experiences, develop an appreciation of others' experiences, and open up new ways of thinking about how to meet challenges that will directly inform co-design.(27) Consistent with the INVOLVE principles for co-producing research,(28) workshops will be co-developed with our Public Involvement group and co-facilitated by an experienced Public Involvement group member.

Potential *public* participants will be invited through existing national PPI networks to join the co-design workshops. Potential *hospice staff and management* participants (clinicians, volunteers, managers, and board members) will be invited through existing communication channels at each site and in consultation with managers. Information will be provided for potential participants with an opportunity to discuss in more detail prior to taking part. Workshops will be scheduled to fit with existing commitments and day-to-day practice at each hospice. PPI team member (MO) will provide input into all aspects of invitations, information provision, and workshop design.

We shall endeavour to maximise diversity within the workshops but acknowledge the tension between attaining diversity across every potential aspect and a maximum workable number of workshop participants of around 15. We shall keep this under review with PPI team member MO.

Central to the conduct of the workshops will be the use of 'touch points' to communicate other peoples' experiences and provide a focus to spark discussion and exploration from different perspectives.(25) Touch points are the events which significantly shape people's positive or negative experience of an event or service. It could be the sharing of a personal or professional experience of delirium care by a workshop participant, or a short film or news item about palliative care services generally or delirium specifically. These will be used to trigger discussion about the detection, assessment, prevention, and management of delirium, how CLECC can be adapted for hospicesand support implementation of delirium guidelines.

Table 3 provides an overview of the schedule and content of the co-design workshops.

For peer review only

Table 3 Co-design workshops schedule and content

Workshop focus	Participants	When, duration	Content
1a. Introduction and initial refinement of CLECC-Pal	Public members	Month 2<br>2 hours	• Introductions<br>• Discussion about the principles of equitable participation<br>• Discussion about the co-design approach to workshops<br>• Introduction to the CLECC strategy and exploration of priority aspects for adaptation<br>• Identification of individual working groups' role in exploring and refining site- or issue-specific aspects of the CLECC strategy before Workshop 2<br>• Agreement on feedback processes outside of the workshops and focus of agenda for Workshop 2
1b. Introduction and initial refinement of CLECC-Pal	Hospice staff and volunteers	Month 2<br>2 hours	As for Workshop 1a
2. Refinement of CLECC-Pal	Public members, hospice staff and volunteers	Month 8<br>3 hours	• Feedback from individual working groups<br>• Discussion of emerging findings from Work Package 3 (realist process evaluation)<br>• Specification of suggested adaptations to CLECC,<br>• Identification of further individual working groups to refine site- or issue-specific aspects of the CLECC strategy<br>• Agreement on focus of agenda for Workshop 3
3. Final specification of CLECC-Pal and celebration	Public members, hospice staff and volunteers	Month 14<br>3 hours	• Feedback from individual working groups<br>• Discussion of further findings from Work Package 3 (realist process evaluation)<br>• Final specification of adaptations to CLECC<br>• Celebration of co-design outputs

For peer review only

Work package 2: Feasibility study

Feasibility will be assessed in the following key areas:

- Patients:
  - Ability to collect high quality, anonymised delirium outcome and process (extent of guideline-adherent care) data from clinical records
  - Variability of baseline delirium day measures to calculate the sample size for a subsequent national study.
- Staff and volunteers: Number of relevant hospice staff and volunteers' participation in CLECC-Pal activities (proportion of relevant staff engaging and maintaining engagement)
- Economic: Ability to collect cost data in relation to CLECC-Pal staff activities

The co-designed CLECC-Pal (for initial version, see Table 1) will be introduced to clinical and support staff, volunteers, and managers at each hospice in a study day that will include training in guideline-recommended delirium care. The study team will support the identified clinical lead to introduce and use CLECC-Pal, including action learning sets, mid-shift 'cluster discussions', twice-weekly reflective discussions and peer observations of practice, over a minimum 12-week period. The study day ethos will emphasise how hospices should take ownership of using CLECC-Pal with only modest support from the study team.

Data collection and analysis

Patients:

Baseline and follow-up (pre and post) clinical record data will be collected. Data will be collected through remote access to the clinical record where electronic records allow, or from the paper record. At each of the three hospices, case note collection (total n=300) will comprise:

- Baseline (pre): 50 consecutive patients who completed their in-patient stay immediately prior to the start of the hospice using CLECC-Pal.
- Follow-up (post): 50 consecutive patients completing their in-patient stay from week 4 of starting use of CLECC-Pal.

Clinical record data collected by the researcher will be anonymised at the point of extraction and include:

- Demographic data (baseline only): age, sex, main medical condition, ethnicity, post code (converted to IMD score)
- Delirium diagnosis using the Inouye et al case note tool(29)
- Delirium management: including evidence of use of delirium screening tools, risk assessments and individualised delirium management care plans

Clinical record data will be extracted using an expanded version of the prospectively validated (74% sensitivity, 83% specificity) chart-based instrument developed by Inouye et al. for detecting potential delirium diagnoses from clinical records.(29) The instrument (data extraction pro-forma, see online supplemental file 1) will enable us to assess whether case-note recorded symptoms of delirium can be linked to time-points during the person's admission when actions around delirium assessment, management and prevention (consistent with guidelines) did or did not take place. Our 'expanded' version of the instrument will include questions about other actions to support delirium assessment, management and prevention that may be recorded in the notes, as shown in Table 4. We shall report the percentage of clinical records where information about each of these actions is recorded. Where a person experiences multiple episodes of delirium within one admission, each episode will be recorded separately and linked through the anonymised case number.

For peer review only

Where judgements about what to record on the pro-forma need to be made, justification for these will be recorded on the form. Any uncertainty about how the information in the case-notes should be recorded on the pro-forma will be discussed with a second clinician (CJ) and justification for the final decision recorded.

Table 4 Additional delirium assessment items to be derived from clinical records and means of assessing feasibility of data collection

Delirium-related action	Assessment of feasibility
Use of Richmond Agitation-Sedation Scale and 4AT screening tools	% completed<br>% re-assessments completed at appropriate timepoints
Medication reviews (to minimise deliriogenic medication)	% completed<br>% re-assessments completed at appropriate timepoints
DSM-V delirium assessment	% completed<br>% re-assessments completed at appropriate timepoints
Degree of sedation or agitation	% completed
Individualised delirium care plans	% completed<br>% reviewed at appropriate timepoints
Presence/absence of delirium	% documenting start and end of delirium episode(s)<br>% documenting delirium-free days

The number of patient records from which it was possible to extract clinical record data *longitudinally* over the duration of their inpatient admission will be reported both as a simple count and as a percentage of the total number of in-patients with a diagnosis of delirium in each hospice each month.

Sample size: Based on our pilot work in one hospice, retrospectively collecting clinical record data for *all* patients whose episode of in-patient care is completed (up to a maximum of 50 per hospice) will provide us with enough data to answer feasibility questions about data quality and enable us to capture frequent events regarding care planning. We do not propose to investigate less-frequent events such as antipsychotic use.

Analysis: Baseline demographic and clinical characteristics of the study population (age, sex, primary medical condition, ethnicity, post code (to derive IMD)) will be presented using descriptive statistics. Mean (SD) will be reported for continuous data and raw count (number, percentage) will be reported for nominal data. The variation around baseline delirium days will be calculated to inform the sample size and number of hospices needed for the subsequent national study.

Staff and volunteers:

In consultation with operational and clinical management at each site, a hospice study lead has been identified through whom the following denominators will be established:

- Number of staff working on or rotating through the in-patient unit of the hospice
- Number of volunteers active within the in-patient unit of the hospice
- Total number of documented in-patient delirium episodes *or* (if total number cannot be established) number of patients with at least one case-note diagnosis of delirium per in-patient admission in the hospice

Level of staff engagement with CLECC-Pal during the implementation period will be assessed weekly by the hospice study lead completing a rapid report of numbers of:

For peer review only

- staff *indirectly* involved in delivering delirium care who attend the team study day, action learning sets, feedback following peer observations of practice, mid-shift cluster discussions, and reflective discussions
- staff and volunteers who do not engage with CLECC-Pal
- staff and volunteers who decrease or stop their engagement with CLECC-Pal
- peer observations of practice achieved
- people approached, reported by professional group and role, who agree to participate in using CLECC-Pal

The rapid report will also record reasons for:

- staff and volunteers' non-engagement or dropout
- modifications made in the use of CLECC-Pal

Quantitative data will be analysed descriptively using radar plots. Qualitative data will be rapidly analysed deductively using a Framework approach.(30) Analyses will inform more detailed exploration in interviews (WP3) and will be shared with participating hospices to inform their ongoing use of CLECC-Pal.

Economic:

We will assess the feasibility of collecting data about the costs of using CLECC-Pal:

- Number of hours spent by members of staff and volunteers in CLECC-Pal activities, linked to pay-grade where possible

Work Package 3: Realist Process Evaluation

Critiques of process evaluations have highlighted the importance of methods that can use theory to explore how contexts and mechanisms interact,(31-33) as recognised in the revised Medical Research Council framework.(34) We shall use realist evaluation(35) to capture staff and management insights into how individual-, team-, and organisational-level contexts affect these interactions during implementation,(36) refining Normalisation Process Theory's (NPT) propositions about the mechanisms of coherence, cognitive participation, collective action, and reflexive monitoring.(37-39). Definitions of realist terms are shown in Table 5. This theoretically-informed understanding of how the implementation strategy *functions*(40) will enable us to explain how hospices may operationalise CLECC-Pal in different ways to achieve the same desired outcomes (for example, by running online learning rather than a team study day, or using self-reflection on practice rather than peer observation).

Table 5 Definition of realist terms used in Work Package 3

Term	Definition
Context	Individual, team, organisational, or other factors that enable or constrain the operation of mechanisms.(41) This includes social phenomena such as rules, norms and values, meaning that contexts are not straightforwardly analogous with settings.(42)
Mechanism	The interaction of a programme's resources or opportunities with individuals' or teams' reasoning.(41)
Outcome	The 'demi-regular' occurrences arising from particular configurations of contexts and mechanisms.(43) Consistent with the recognition in realist ontology of the dynamic and non-linear nature of open systems

For peer review only

	in the social world,(44) ‘outcomes’ may be better understood as semi-stable processes.
Programme Theory	A middle-range theoretical explanation of how (implementation) programme activities relate to underlying theory. Even if not explicitly stated, programme theories contain ideas about how best to address challenges to achieving intended goals (including how to proactively manage these challenges)(45)

Identification, sampling and consent

Surveys

All hospice staff involved in direct patient care or management, as well as those directly involved in patient care (volunteers, support staff, board members with a hospice governance role) will be eligible. Minimum sample size of 10 at each hospice (total n=30). Eligible participants will be sent a link to the anonymous survey, for which completion online will be taken as implied consent.

Interviews

A purposive sampling strategy at each site will draw from a sampling frame that includes all hospice staff involved in direct patient care or management, volunteers, support staff, and board members at each study site. Within the constraints of an exploratory sample size (five staff and volunteers, and two members of management and/or executive board at each site; minimum total n=15), we shall endeavour to maximise variation in participant characteristics and roles, prioritising sampling that will enable comparison between those who do and do *not* take part. Written informed consent will be obtained. Interviews will be conducted at a time suitable for participants and may be face-to-face or remote, according to participant preference.

Data collection and analysis

Staff and volunteers’ pre- and post-implementation experiences (survey):

Survey using a modified and piloted Normalisation Measurement Instrument (NoMad).(46) of staff and volunteers’ perceptions and experiences of implementation, in relation to each NPT mechanism, before and after using the CLECC-Pal implementation strategy.

Quantitative Likert scale responses will be analysed descriptively using radar plots. Free-text responses will be deductively thematically-analysed using the framework of NPT mechanisms (coherence, cognitive participation, collective action, and reflexive monitoring), allowing for inductive thematic analysis if responses do not fit within the framework. Thematic patterns and outliers will be identified. The analysis will also inform the structure, content, and focus of the staff and volunteer interviews.

Staff and volunteers’ post-implementation experiences (interviews):

Realist interviews are distinct from conventional qualitative semi-structured interviews as they adopt a ‘teacher-learner’ approach. This involves presenting theory to participants so that they can communicate their own experiences and views that may refute, refine, or expand the theory.(47) In practice, the realist interviewer presents theory (context-mechanism-outcome configurations) in a form comprehensible to the participant and follows-

For peer review only - http://bmjopen.bmj.com/site/about/guidelines.xhtml

up flexibly with further questions tailored to the participant's understanding, to ensure that the discussion enables theory-refinement rather than simply a discussion of experiences. Interviews will build on Murray et al's.(48) operationalisation of NPT for the development and optimisation of interventions within trials (see Table 6).

Interview topics will include, but not be limited to, experiences of CLECC-Pal's acceptability and fit, rationale for any modifications to CLECC-Pal, perceived changes in communication between those caring for patients at-risk of delirium, changes in care practices, perceptions about how CLECC-Pal is achieving (or not) the intended effects and, if appropriate, how these impacts could be sustained. Interview questions will be informed by emerging site-specific data from the co-design and feasibility work packages, as well as from the process evaluation survey. Graphical summaries of data, such as radar plots, will be used in the interviews to communicate this emerging data to participants, link to theory, and to support discussion that enables implementation theory to be refined.(47, 49) Views of study processes will also be sought. It is envisaged that interviews will last no longer than 30 minutes, but participants will be given the opportunity for a longer interview if they wish.

Interviews will be recorded and transcribed. Before commencing analysis, interview transcripts will be read and re-read to allow familiarisation with the content that will enable theory-building and refinement rather than rote coding of contexts, mechanisms and outcomes (although coding of these configurations may also play an important role in theory-building and refinement). Analysis to identify contextualised explanations of how mechanisms of implementation are understood to lead to certain outcomes will be structured using the reasoning processes identified by Pawson (juxtaposition, reconciliation, adjudication, consolidation, and situating(50)). We shall operationalise these reasoning processes using the analytic questions for building and refining programme theory identified by Pearson et al.(51)

Work Package 3 methods and findings will be reported consistent with the RAMESES reporting standards.(43)

For peer review only

Table 6 Normalisation Process Theory 'Contribution' mechanisms and their relationship to data collection in interviews

Mechanism	Definition(37)	Theoretical propositions(38)	Potential interview questions(48)
1.Coherence	Agents attribute meaning to a complex intervention and make sense of its possibilities within their field of agency. They frame how participants make sense of, and specify, their involvement in a complex intervention.	1.1 Embedding is dependent on work that defines and organises a practice as a cognitive and behavioural ensemble.<br>1.2 Embedding work is shaped by factors that promote or inhibit actors' apprehension of a practice as meaningful.<br>1.3 The production and reproduction of coherence in a practice requires that actors collectively invest meaning in it.	Is CLECC-Pal:<br>- easy to describe?<br>- clearly distinct from other strategies?<br>- have a clear purpose for all participants?<br>Do participants have a shared sense of purpose?<br>What benefits will the intervention bring and to whom?<br>Are these benefits likely to be valued by potential participants?<br>Will CLECC-Pal fit with the overall goals and activity of the organisation?
2.Cognitive Participation	Agents legitimise and enrol themselves and others into a complex intervention. They frame how participants become members of a specific community of practice.	2.1 Embedding is dependent on work that defines and organises the actors implicated in a practice.<br>2.2 Embedding work is shaped by factors that promote or inhibit actors' participation.<br>2.3 The production and reproduction of a practice requires that actors collectively invest commitment in it.	Are target user groups likely to think that CLECC-Pal is a good idea?<br>Will they see the point of CLECC-Pal?
3.Collective Action	Agents mobilise skills and resources and enact a complex intervention. They frame how participants realise and perform the intervention in practice.	3.1 Embedding is dependent on work that defines and operationalises a practice.<br>3.2 Embedding work is shaped by factors that promote or inhibit actors' enacting it.<br>3.3 The production and reproduction of a practice requires that actors collectively invest effort in it.	How will CLECC-Pal affect the work of user groups?<br>Will CLECC-Pal promote or impede their work?<br>Will staff require extensive training before they can use CLECC-Pal?<br>How compatible with existing work practices is CLECC-Pal?<br>What impact will CLECC-Pal have on division of labour, resources, power, and responsibility between different professional groups?<br>Will CLECC-Pal fit with the overall goals and activity of the organisation?

For peer review only

Mechanism	Definition(37)	Theoretical propositions(38)	Potential interview questions(48)
4.Reflexive Monitoring	Agents assemble and appraise information about the effects of a complex intervention within their field of agency, and utilise that knowledge to reconfigure social relations and action. They frame how participants collect and utilise information about the effects of the intervention.	4.1 Embedding is dependent on work that defines and organises the everyday understanding of a practice.<br>4.2 Embedding work is shaped by factors that promote or inhibit appraisal.<br>4.2 The production and reproduction of a practice requires that actors collectively invest in its understanding.	How are users likely to perceive CLECC-Pal once it has been used for a while?<br>Is CLECC-Pal likely to be perceived as advantageous for patients or staff?<br>Will it be clear what effects CLECC-Pal has had?<br>Can users contribute feedback about CLECC-Pal once it is in use?<br>Can CLECC-Pal be adapted or improved on the basis of experience?

For peer review only

Ethics and dissemination

Ethical approval for the study has been obtained from Hull York Medical School Ethics Committee (Ref.: 21/23), Health Research Authority Research Ethics Committee Wales REC7 (Ref.: 21/WA/0180) and Health Research Authority Confidentiality Advisory Group (Ref.: 21/CAG/0071). Confidentiality Advisory Group approval allows the study researcher access to the clinical records to extract data without patient consent. The study is publicised in the hospices during the data collection period and patients/representatives may opt out if they do not wish their data to be used. Written informed consent will be obtained from interview participants.

he primary objective of this study is to inform a future quasi-experimental multi-site comparative evaluation. We shall do this by demonstrating the feasibility (or otherwise) of the implementation strategy ('intervention'), participant recruitment, and data collection, in addition informing decisions about the most appropriate study design for a future multi-site comparative evaluation. However, as argued by Thabane et al.,(52) communicating findings from feasibility studies remains critically important for ensuring that resources are not spent on either duplicating the feasibility study or funding research uninformed by the findings of a relevant feasibility study. We shall therefore prepare a full report of the study's methods and findings for the funder and submit a manuscript reporting the findings to an open access peer-reviewed journal. The study's findings will also be submitted for oral presentation at one national health services research conference and one international palliative care conference. A Plain English summary of study findings will be prepared for distribution through palliative care clinical networks (including Hospice UK) and Public Involvement groups.

Discussion

This study will address key uncertainties about the implementation of guideline-adherent delirium care in hospices - the feasibility of: using a theoretically-informed, co-developed implementation strategy (CLECC-Pal); collecting demographic, diagnostic, and delirium management data from clinical records; collecting measures of staff engagement; and collecting explanatory process data about staff use of CLECC-Pal. This will enable us to estimate the number of hospice sites and in-patient episodes needed for the planned national quasi-experimental study, for which we outline the design considerations below. The study has clear strengths in public involvement and in minimising research waste by using existing process and outcome data. There are also limitations in the study, for example, hospices are all drawn from a single region of the United Kingdom and the sample size for surveys and interviews may limit the extent to which the complexity of staff and management characteristics, views and experiences can be explored. Nevertheless, the study hospices have diverse characteristics (locations, level of socio-economic deprivation, forms of governance) and we shall purposively sample staff and management (for interviews) to maximise the range of professional and role characteristics.

We have developed this Feasibility study to inform future decisions about evaluative study design that balances scientific rigour and practical considerations. In doing so, we first appraised an interrupted time series design that would enable naturalistic data collection, but considered this unrealistic as powering the study would likely require 12 months pre- and post-intervention data collection.(53) Second, we appraised a randomised stepped wedge design, but considered implementation research permutations of this design unlikely to be

For peer review only

feasible due to the real-world setting (if using a head-to-head rollout design) or length of time required (if using a pairwise enrolment rollout design).(54)

Consistent with current thinking in implementation research for investigators to consider quasi-experimental study designs that can assess the impact of context over time(55), we plan to work towards an evaluative study design that uses natural variation in the introduction of the implementation strategy to allow a *non-randomised* stepped wedge design (CLECC-Pal supported delirium care vs. delirium care as usual). Our audit data indicate that this would be realistic given an annual admission rate of 192-384 in the 10-20 bedded study site hospices which have a 40-60% incidence of delirium.

Whilst hospices are relatively homogeneous in terms of care delivery by health professionals (e.g. standardised national training programme for doctors, national standards for nursing practice), the wide referral base of hospices mean that in-patients tend to be heterogeneous in relation to type and stage of disease, ethnicity, socio-economic status, and so on. For the future evaluative study, we shall estimate the intraclass correlation coefficient (ICC) using pre-intervention patient outcome data (delirium-free days) from the feasibility study, thus enabling a sample size calculation powered on the primary outcome for the future evaluative study.

We are mindful of a recent systematic review of feasibility studies which identified a lack of consistency in the use of terminology, a predominance of feasibility issues relating to preparation for randomised-controlled trials, and an absence of clear guidance about when “sufficient insight about uncertainties” had been achieved for progression to an evaluation study.(56, p.10) However, we are confident in stating minimum recruitment targets for the use of CLECC-Pal (fidelity to core components) and 4AT screening tool at baseline and daily, that will be necessary for a future evaluative study to be considered feasible:

- ≥80%, proceed
- 60 – 80% with mitigating factors, proceed
- <60% not feasible

Author contributions: MP and MJ led study conceptualisation and design, with contributions from CJ, JB and NS. MP and MJ led development of analysis plans, with contributions from CJ, CH and MT. MP led the writing process and drafted the original protocol with input from GJ and MJ. Critical review of the protocol and contributions to refinement to: co-design work package from GJ, MO, IF and MT; feasibility work package from GJ, CJ, JB, IF, CH, MO, KS, NS, MT and MJ; process evaluation work package from GJ, CJ, IF, MT and MJ. All authors take responsibility for the protocol and approved the final version of this paper.

Funding statement: This work is supported by Yorkshire Cancer Research (Award reference number HEND405DEL).

Competing interests statement: The authors declared no potential conflicts of interest with respect to the research, authorship, and/or publication of this article.

Acknowledgements: Creating Learning Environments for Compassionate Care (CLECC) © 2020 University of Southampton. Used under non-exclusive licence.

For peer review only

Figures:

Figure 1 Study flowchart and timeline summary

References:

1. American Psychiatric Association. Diagnostic and Statistical Manual of Mental Disorders, Fifth Edition (DSM-5). Arlington, VA: American Psychiatric Publisher; 2013.
2. Hosie A, Davidson PM, Agar M, Sanderson CR, Phillips J. Delirium prevalence, incidence, and implications for screening in specialist palliative care inpatient settings: A systematic review. Palliative Medicine. 2012;27:486–98.
3. Brajtman S, Higuchi K, McPherson C. Caring for patients with terminal delirium: palliative care unit and home care nurses' experiences. International Journal of Palliative Nursing. 2006;12(4):150-6.
4. Fang CK, Chen HW, Liu SI, Lin CJ, Tsai LY, Lai YL. Prevalence, detection and treatment of delirium in terminal cancer inpatients: a prospective survey. Jpn J Clin Oncol. 2008;38(1):56-63.
5. Lawlor PG, Gagnon B, Mancini IL, Pereira JL, Hanson J, Suarez-Almazor ME, et al. Occurrence, causes, and outcome of delirium in patient with advanced cancer: a prospective study. Arch Intern Med. 2000;160(6):786-94.
6. Weinrebe W, Johannsdottir E, Karaman M, Fusgen I. What does delirium cost? An economic evaluation of hyperactive delirium. Z Gerontol Geriatr. Z Gerontol Geriatr. 2016;49(1):52-8.
7. Zaubler TS, Murphy K, Rizzuto L, Santos R, Skotzko C, Giordano J, et al. Quality improvement and cost savings with multicomponent delirium interventions: replication of the Hospital Elder Life Program in a community hospital. Psychosomatics. 2013;54(3):219-26.
8. Watt CL, Momoli F, Ansari MT, Sikora L, Bush SH, Hosie A, et al. The incidence and prevalence of delirium across palliative care settings: A systematic review. Palliative Medicine. 2019;DOI: 10.1177/0269216319854944.
9. Oh ES, Fong TG, Hshieh TT, Inouye SK. Delirium in older persons: Advances in diagnosis and treatment. JAMA. 2017;318(12):1161-74.
10. Thom RP, Levy-Carrick NC, Bui M, Silbersweig D. Delirium. American Journal of Psychiatry. 2019;176(10):785-93.
11. Leslie DL, Inouye SK. The importance of delirium: economic and societal costs. J Am Geriatr Soc. 2011;59 (Suppl 2):S241-3.
12. NICE. Delirium: prevention, diagnosis and management - Clinical Guideline 103. London: National Institute for Health & Care Excellence; 2010.
13. Scottish Intercollegiate Guidelines Network. Risk reduction and management of delirium: A national clinical guideline. Edinburgh: NHS Scotland; 2019.
14. Australian Commission on Safety and Quality in Health Care. Delirium Clinical Care Standard. Sydney: ACSQHC; 2016.
15. Featherstone I, Hosie A, Siddiqi N, Grassau P, Bush SH, Taylor J, et al. The experience of delirium in palliative care settings for patients, family, clinicians and volunteers: A qualitative systematic review and thematic synthesis. Palliative Medicine. 2021;35(6):988-1004.
16. Boland JW, Kabir M, Bush SH, Spiller JA, Johnson MJ, Agar M, et al. Delirium management by palliative medicine specialists: a survey from the association for palliative medicine of Great Britain and Ireland. BMJ Supportive & Palliative Care. 2019.
17. Woodhouse R, Siddiqi N, Boland JW, Featherstone I, Johnson MJ. Screening for delirium: A survey of delirium screening practice in specialist palliative care units in the UK. BMj Supportive & Palliative Care. 2020;[Submitted for publication].
18. Steinhauser KE, Christakis NA, Clipp EC. Factors Considered Important at the End of Life by Patients, Family, Physicians, and Other Care Providers. JAMA. 2000;284(19):2476-82.
19. Hosie A, Lobb E, Agar M, Davidson PM, Phillips J. Identifying the barriers and enablers to palliative care nurses' recognition and assessment of delirium symptoms: a qualitative study. Journal of Pain & Symptom Management. 2014;48(5):815-30.
20. Bridges J, Pickering RM, Barker H, Chable R, Fuller A, Gould L, et al. Implementing the Creating Learning Environments for Compassionate Care (CLECC) programme in acute hospital settings: a pilot RCT and feasibility study. Health Serv Deliv Res. 2018;6(33).
21. Bridges J, Gould L, Libberton P, Schoonhoven L. Creating learning environments for compassionate care: a programme to support nursing leaders and teams to deliver compassionate hospital care - Manual for Practice Development Leads. Southampton: University of Southampton; 2020.

For peer review only

22. Hoffmann TC, Glasziou PP, Boutron I, Milne R, Perera R, Moher D, et al. Better reporting of interventions: template for intervention description and replication (TIDieR) checklist and guide. BMJ. 2014;348:g1687.
23. UK Public Involvement Standards Development Partnership. UK Standards for Public Involvement. London: NIHR Central Commissioning Facility; 2019.
24. Bate P, Robert G. Experience-based design: from redesigning the system around the patient to co-designing services with the patient. 2006;15(5):307-10.
25. Locock L, Robert G, Boaz A, Vougioukalou S, Shuldham C, Fielden J, et al. Testing accelerated experience-based co-design: a qualitative study of using a national archive of patient experience narrative interviews to promote rapid patient-centred service improvement. Health Serv Deliv Res. 2014;2(4).
26. Pearson N, Naylor P-J, Ashe MC, Fernandez M, Yoong SL, Wolfenden L. Guidance for conducting feasibility and pilot studies for implementation trials. Pilot and Feasibility Studies. 2020;6(167).
27. Iedema R, Merrick E, Piper D, Britton K, Gray J, Verma R, et al. Codesigning as a discursive practice in emergency health services: the architecture of deliberation. Journal of Applied Behavioral Science. 2010;46(1):73-91.
28. INVOLVE. Guidance on co-producing a research project. Southampton: INVOLVE; 2018.
29. Inouye SK, Leo-Summers L, Zhang Y, Bogardus ST, Leslie DL, Agostini JV. A chart-based method for identification of delirium: validation compared with interviewer ratings using the Confusion Assessment Method. J Am Geriatr Soc. 2005;53:312-8.
30. Ritchie J, Lewis J, McNaughton-Nicholls C, Ormston R, editors. Qualitative Research Practice: A guide for social science students and researchers. 2nd edition ed. London: Sage; 2013.
31. Moore GF, Audrey S, Barker M, Bond L, Bonell C, Hardeman W, et al. Process evaluation of complex interventions: Medical Research Council guidance. BMJ. 2015;350:h1258.
32. Murdoch J. Process evaluation for complex interventions in health services research: analysing context, text trajectories and disruptions. BMC Health Serv Res. 2016;16(407).
33. Wells M, Williams B, Treweek S, Coyle J, Taylor J. Intervention description is not enough: evidence from an in-depth multiple case study on the untold role and impact of context in randomised controlled trials of seven complex interventions. Trials. 2012;13:95-111.
34. Skivington K, Matthews L, Simpson SA, Craig P, Baird J, Blazeby JM, et al. A new framework for developing and evaluating complex interventions: update of Medical Research Council guidance. BMJ. 2021;374:n2061.
35. Pawson R. The Science of Evaluation: A Realist Manifesto. London: Sage; 2013.
36. O'Brien R, Buston K, Wight D, McGee E, White J, Henderson M. A realist process evaluation of Enhanced Triple P for Baby and Mellow Bumps, within a Trial of Healthy Relationship Initiatives for the Very Early years (THRIVE): study protocol for a randomized controlled trial. Trials. 2019;20(1):351.
37. May C. Towards a general theory of implementation. Implementation science : IS. 2013;8:18.
38. May C, Finch T. Implementing, Embedding, and Integrating Practices: An Outline of Normalization Process Theory. Sociology. 2009;43(3):535-54.
39. May CR, Cummings A, Girling M, Bracher M, Mair FS, May CM, et al. Using Normalization Process Theory in feasibility studies and process evaluations of complex healthcare interventions: a systematic review. Implementation science : IS. 2018;13(1):80.
40. Hawe P, Shiell A, Riley T. Theorising interventions as events in systems. American journal of community psychology. 2009;43(3-4):267-76.
41. Pearson M, Chilton R, Wyatt K, Abraham C, Ford T, Woods HB, et al. Implementing health promotion programmes in schools: a realist systematic review of research and experience in the United Kingdom. 2015;10(1):149.
42. Pawson R, Tilley N. Realistic Evaluation. London: Sage Publications; 1997.
43. Wong G, Westhorp G, Manzano A, Greenhalgh J, Jagosh J, Greenhalgh T. RAMESES II reporting standards for realist evaluations. BMC medicine. 2016;14(1):96.
44. Sayer A. Realism and Social Science. London: Sage; 2000.
45. Pawson R, Owen L, Wong G. The Today Programme's contribution to Evidence-Based Policy. Evaluation. 2010;16(2):211-3.
46. Finch TL, Girling M, May CR, Mair FS, Murray E, Treweek S, et al. Nomad: Implementation measure based on Normalization Process Theory. [Measurement instrument]. 2015 [01/12/2021]. Available from: .
47. Manzano A. The craft of interviewing in realist evaluation. Evaluation. 2016;22(3):342-60.

For peer review only

48. Murray E, Treweek S, Pope C, MacFarlane A, Ballini L, Dowrick C, et al. Normalisation process theory: a framework for developing, evaluating and implementing complex interventions. . 2010;8(63).
49. Mukumbang FC, Marchal B, Van Belle S, van Wyk B. Using the realist interview approach to maintain theoretical awareness in realist studies. Qualitative Research. 2019:146879411988198.
50. Pawson R. Evidence-Based Policy: A Realist Perspective. London: Sage Publications; 2006.
51. Pearson M, Brand SL, Quinn C, Shaw J, Maguire M, Michie S, et al. Using realist review to inform intervention development: methodological illustration and conceptual platform for collaborative care in offender mental health. 2015;10(1):134.
52. Thabane L, Ma J, Chu R, Cheng J, Ismaila A, Rios LP, et al. A tutorial on pilot studies: the what, why and how. BMC Medical Research Methodology. 2010;10(1).
53. Zhang F, Wagner AK, Ross-Degnan D. Simulation-based power calculation for designing interrupted time series analyses of health policy interventions. Journal of Clinical Epidemiology. 2011;64:1252-61.
54. Brown CH, Curran G, Palinkas LA, Aarons GA, Wells KB, Jones L, et al. An Overview of Research and Evaluation Designs for Dissemination and Implementation. Annu Rev Public Health. 2017;38:1-22.
55. Kemp CG, Wagenaar BH, Haroz EE. Expanding Hybrid Studies for Implementation Research: Intervention, Implementation Strategy, and Context. Frontiers in Public Health. 2019;7.
56. Hallingberg B, Turley R, Segrott J, Wight D, Craig P, Moore L, et al. Exploratory studies to decide whether and how to proceed with full-scale evaluations of public health interventions: a systematic review of guidance. Pilot and Feasibility Studies. 2018;4(104).

For peer review only

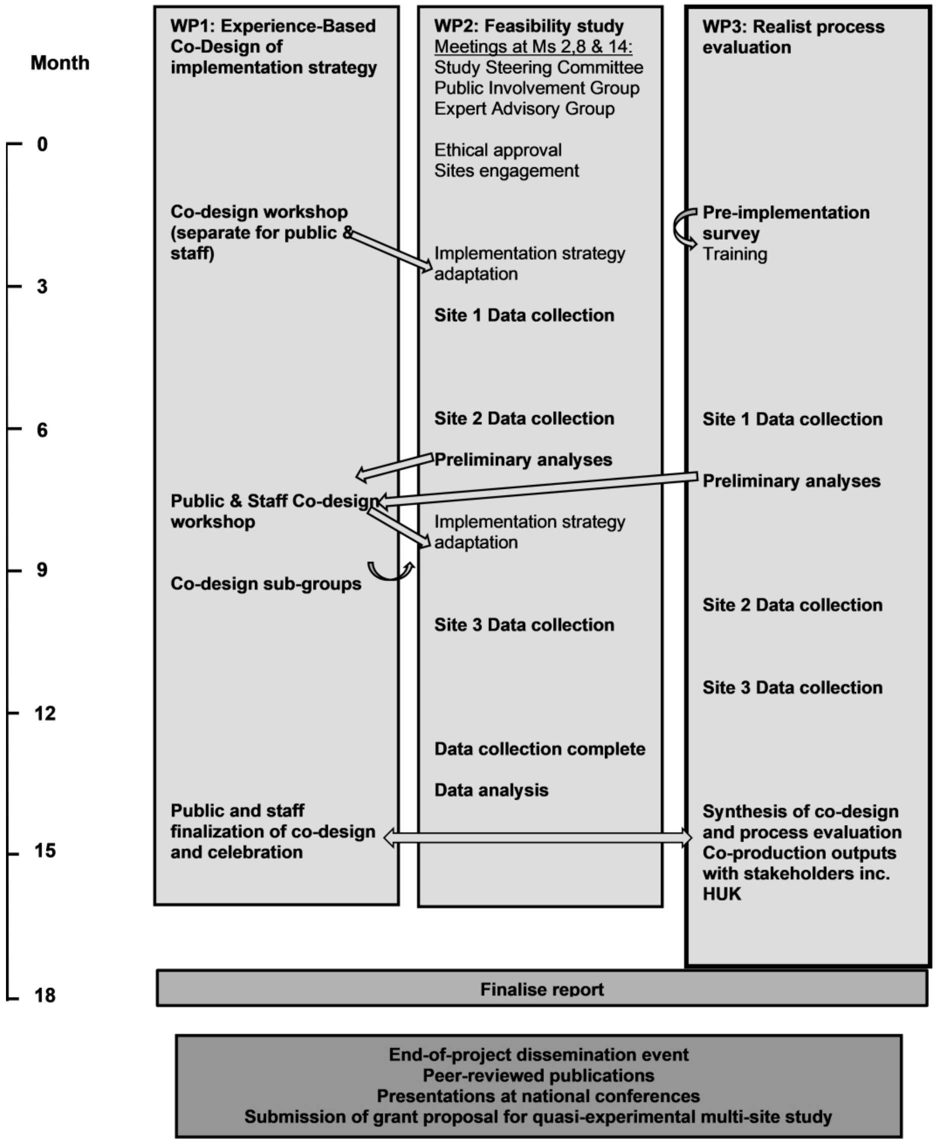

Figure 1 Study flowchart and timeline summary

1034x1326mm (96 x 96 DPI)

Online supplemental file 1
'Improving the Detection, Assessment, Management, and Prevention of Delirium in Hospices (the DAMPen-D study): Protocol for a co-design and feasibility study of a flexible and scalable implementation strategy to deliver guideline-adherent delirium care.'

**Case Number_Pre**	Non-identifiable ID number	N/A	Alphnumeric
**Age_Pre**	Patient age	Integer	Years
**Sex_Pre**	Patient gender	Categorical	Male, female, other
**Diagnosis_Pre**	Patient diagnosis	String	Cancer etc.
**Ethnicity_pre**	Patient ethnicity	Categorical	Black, white etc.
**IMD score_Pre**	Postcode converted	Float	
**Adm_Ac_conf_state_Pre**	Evidence of acute confusional state on admission	Binary	Yes/No
**Adm_screen_Pre**	Patient screened for delirium on admission	Binary	Yes/No
**Adm_Screen_by_Pre**	If screened, who completed screening	Categorical	Doctor, Nurse practitioner, Registered Nurse, Other (specify)
**Adm_screen_type_Pre**	If screened, name of screening tool	Alphanumeri c	4AT etc.
**Adm_screen_result_Pre**	If screened, result	Binary	Positive/Negative
**Adm_no_screen_just-Pre**	Was justification given for not screening	Binary	Yes/No
**Adm_no-screen_just-verbatim-Pre**	Was justification given for not screening	String	Verbatim text

For peer review only - http://bmjopen.bmj.com/site/about/guidelines.xhtml

Online supplemental file 1
'Improving the Detection, Assessment, Management, and Prevention of Delirium in Hospices (the DAMPen-D study): Protocol for a co-design and feasibility study of a flexible and scalable implementation strategy to deliver guideline-adherent delirium care.'

**Adm_risk-ass-Pre**	If screening negative or not done was risk assessment carried out?	Binary	Yes/No
**Adm-risk-ass_result_Pre**	If risk assessment carried completed results	Binary	Positive/Negative
**Adm_prev_meas_Pre**	If risk assessment positive were preventive measures put in place	Binary	Yes/No
**Adm_Judge_rationale_pre**	If researcher judgement was required for any of above, give rationale	String	Free text
**Dur_adm_Ac_conf_state_pre**	Evidence of acute confusional state during admission	Binary	Yes/No
**Dur_adm_ Multi_ep_cog_dys_Pre**	Multiple episodes of cognitive dysfunction during admission	Binary	Yes/No
**Dur_adm_ Multi_ep_cog_dys_no_Pre**	Multiple episodes of cognitive dysfunction during admission	Integer	1,2,3

For peer review only - http://bmjopen.bmj.com/site/about/guidelines.xhtml

Online supplemental file 1
'Improving the Detection, Assessment, Management, and Prevention of Delirium in Hospices (the DAMPen-D study): Protocol for a co-design and feasibility study of a flexible and scalable implementation strategy to deliver guideline-adherent delirium care.'

**Dur_adm_screen_Pre**	If patient had multiple episodes was the patient screened	Binary	Yes/No
**Dur_adm_screen_type_Pre**	If screened, name of tool	Alphanumeri c	4AT etc.
**Dur_Adm_Screen_result_Pre**	Result of screening during admission	Binary	Positive/negative
**Dur_adm_Sceen_by_Pre**	Who completed screening during admission	Categorical	Doctor, Nurse practitioner, Registered Nurse, Other (specify)
**Dur_adm_Judge_rationale_Pre**	If researcher judgement was required for any of above, give rationale	String	Verbatim text
**Case_rec_ac_conf_reported_by_Pr e**	Who reported the first episode of acute confusion in the case record	Categorical	Doctor, Nurse practitioner, Registered Nurse, Other (specify)
**Case_rec_date_first_ep_Pre**	Date of first episode of acute confusion in the case record	Date	10.10.2021
**Case_rec_time_first_ep_Pre**	Time of first episode of acute confusion in case record	Time	24hr format

For peer review only - http://bmjopen.bmj.com/site/about/guidelines.xhtml

Online supplemental file 1
'Improving the Detection, Assessment, Management, and Prevention of Delirium in Hospices (the DAMPen-D study): Protocol for a co-design and feasibility study of a flexible and scalable implementation strategy to deliver guideline-adherent delirium care.'

**Case_rec_verbatim_ref_ac_conf_Pre**	Describe each reference to acute confusion in the case record	String	Verbatim text
**Case_rec_ac_conf_tot_days_Pre**	Total duration of acute confusion in days as determined by all the references in the case record	Integer	5 (days) or 0 days if none
**Case_rec_Improve_revers_Pre**	Any evidence of improvement or reversibility of acute confusion during the stay	Categorical	Yes/No/Unsure
**Case_rec_ev_descr_pre**	Describe evidence of reversibility	String	Free text
**Case_rec_Del_present_Pre**	Delirium present	Categorical	Yes/No
**Case_rec_subtype_Pre**	If delirium present what subtype	Categorical	Hypo/Hyper/Mixed
**Case_rec_del_med_ass_Pre**	Medical assessment (DSM-V delirium assessment) to assess for delirium	Binary	Yes/No
**Case_rec_diag_doc_Pre**	Diagnosis of delirium recorded	Categorical	Yes/No

For peer review only - http://bmjopen.bmj.com/site/about/guidelines.xhtml

Online supplemental file 1
'Improving the Detection, Assessment, Management, and Prevention of Delirium in Hospices (the DAMPen-D study): Protocol for a co-design and feasibility study of a flexible and scalable implementation strategy to deliver guideline-adherent delirium care.'

**Case_rec_judge_rationale_Pre**	If researcher judgement was required for any of above, give rationale	String	Free text
**Invest_del_ ass_rev_cause_Pre**	Assessment for reversible causes of delirium	Binary	Yes/No
**Invest_med_rev_Pre**	Was a medication review conducted	Binary	Yes/No
**Invest_rev_cause_treat_Pre**	Was a treatment instigated for a reversible cause of delirium	Binary	Yes/No
**Invest_ judge_rationale_Pre**	If researcher judgement was required for any of above, give rationale	String	Free text
**Del_care_plan_Pre**	Delirium care plan documented	Binary	Yes/no
**Del_sev_Pre**	Was delirium severity assessed	Categorical	RASS-PAL + hallucination,RASS-PAL only, hallucination only, other specify, No

For peer review only - http://bmjopen.bmj.com/site/about/guidelines.xhtml

Online supplemental file 1
'Improving the Detection, Assessment, Management, and Prevention of Delirium in Hospices (the DAMPen-D study): Protocol for a co-design and feasibility study of a flexible and scalable implementation strategy to deliver guideline-adherent delirium care.'

**Harm_distress_behaviour_Pre**	Did patient display behaviours harmful or distressing to self or others	Binary	Yes/No
**Sedative_admin_during_del_Pre**	Was sedative administered during period of delirium	Binary	Yes/No
**Sedative_med_type**	Sedative medication type	String	Name of medication
**Sed_ind_Pre**	Sedative medication administered for	Categorical	Delirium, anxiety, breathlessness, nausea, terminal agitation, other, unclear
**Del_risk_discuss_patient_fam**	Was delirium risk and prevention discussed with patients and families of patients without delirium on admission	Categorical	Yes/No/unable
**Del_ep_discuss_patient_Pre**	Was episode of delirium discussed with the patient	Categorical	Yes/No/Unable

For peer review only - http://bmjopen.bmj.com/site/about/guidelines.xhtml

Online supplemental file 1
'Improving the Detection, Assessment, Management, and Prevention of Delirium in Hospices (the DAMPen-D study): Protocol for a co-design and feasibility study of a flexible and scalable implementation strategy to deliver guideline-adherent delirium care.'

**Del_ep_discuss_patient_family_Pre**	Was episode of delirium discussed with the patient's family	Categorical	Yes/No/Unable
**Del_info_Pre**	Was any written information about delirium provided to patient or family	Categorical	Yes/No/Unable

For peer review only

CONSORT 2010 checklist of information to include when reporting a pilot or feasibility trial*

Section/Topic	Item No	Checklist item	Reported on page No
**Title and abstract**			
	1a	Identification as a pilot or feasibility randomised trial in the title	1
	1b	Structured summary of pilot trial design, methods, results, and conclusions (for specific guidance see CONSORT abstract extension for pilot trials)	2
**Introduction**			
Background and objectives	2a	Scientific background and explanation of rationale for future definitive trial, and reasons for randomised pilot trial	3-4
	2b	Specific objectives or research questions for pilot trial	4, 6
**Methods**			
Trial design	3a	Description of pilot trial design (such as parallel, factorial) including allocation ratio	NA (protocol)
	3b	Important changes to methods after pilot trial commencement (such as eligibility criteria), with reasons	NA (protocol)
Participants	4a	Eligibility criteria for participants	9
	4b	Settings and locations where the data were collected	4
	4c	How participants were identified and consented	9, 12, 15
Interventions	5	The interventions for each group with sufficient details to allow replication, including how and when they were actually administered	5
Outcomes	6a	Completely defined prespecified assessments or measurements to address each pilot trial objective specified in 2b, including how and when they were assessed	9-11, 12
	6b	Any changes to pilot trial assessments or measurements after the pilot trial commenced, with reasons	NA (protocol)
	6c	If applicable, prespecified criteria used to judge whether, or how, to proceed with future definitive trial	16
Sample size	7a	Rationale for numbers in the pilot trial	9-10
	7b	When applicable, explanation of any interim analyses and stopping guidelines	NA
Randomisation:			
Sequence generation	8a	Method used to generate the random allocation sequence	NA
	8b	Type of randomisation(s); details of any restriction (such as blocking and block size)	NA
Allocation concealment mechanism	9	Mechanism used to implement the random allocation sequence (such as sequentially numbered containers), describing any steps taken to conceal the sequence until interventions were assigned	NA

For peer review only

Implementation	10	Who generated the random allocation sequence, who enrolled participants, and who assigned participants to interventions	NA
Blinding	11a	If done, who was blinded after assignment to interventions (for example, participants, care providers, those assessing outcomes) and how	NA
	11b	If relevant, description of the similarity of interventions	NA
Statistical methods	12	Methods used to address each pilot trial objective whether qualitative or quantitative	10, 12
**Results**			
Participant flow (a diagram is strongly recommended)	13a	For each group, the numbers of participants who were approached and/or assessed for eligibility, randomly assigned, received intended treatment, and were assessed for each objective	NA (protocol)
	13b	For each group, losses and exclusions after randomisation, together with reasons	NA (protocol)
Recruitment	14a	Dates defining the periods of recruitment and follow-up	NA (protocol)
	14b	Why the pilot trial ended or was stopped	NA (protocol)
Baseline data	15	A table showing baseline demographic and clinical characteristics for each group	NA (protocol)
Numbers analysed	16	For each objective, number of participants (denominator) included in each analysis. If relevant, these numbers should be by randomised group	NA (protocol)
Outcomes and estimation	17	For each objective, results including expressions of uncertainty (such as 95% confidence interval) for any estimates. If relevant, these results should be by randomised group	NA (protocol)
Ancillary analyses	18	Results of any other analyses performed that could be used to inform the future definitive trial	NA (protocol)
Harms	19	All important harms or unintended effects in each group (for specific guidance see CONSORT for harms)	NA (protocol)
	19a	If relevant, other important unintended consequences	NA (protocol)
**Discussion**			
Limitations	20	Pilot trial limitations, addressing sources of potential bias and remaining uncertainty about feasibility	NA (protocol)
Generalisability	21	Generalisability (applicability) of pilot trial methods and findings to future definitive trial and other studies	16
Interpretation	22	Interpretation consistent with pilot trial objectives and findings, balancing potential benefits and harms, and considering other relevant evidence	NA (protocol)
	22a	Implications for progression from pilot to future definitive trial, including any proposed amendments	16
**Other information**			
Registration	23	Registration number for pilot trial and name of trial registry	2
Protocol	24	Where the pilot trial protocol can be accessed, if available	NA (protocol)
Funding	25	Sources of funding and other support (such as supply of drugs), role of funders	17
	26	Ethical approval or approval by research review committee, confirmed with reference number	16

For peer review only

Citation: Eldridge SM, Chan CL, Campbell MJ, Bond CM, Hopewell S, Thabane L, et al. CONSORT 2010 statement: extension to randomised pilot and feasibility trials. BMJ. 2016;355.

*We strongly recommend reading this statement in conjunction with the CONSORT 2010, extension to randomised pilot and feasibility trials, Explanation and Elaboration for important clarifications on all the items. If relevant, we also recommend reading CONSORT extensions for cluster randomised trials, non-inferiority and equivalence trials, non-pharmacological treatments, herbal interventions, and pragmatic trials. Additional extensions are forthcoming: for those and for up to date references relevant to this checklist, see www.consort-statement.org.

For peer review only

BMJ Open

Improving the Detection, Assessment, Management, and Prevention of Delirium in Hospices (the DAMPen-D study): protocol for a co-design and feasibility study of a flexible and scalable implementation strategy to deliver guideline-adherent delirium care

Journal:	*BMJ Open*
Manuscript ID	bmjopen-2021-060450.R2
Article Type:	Protocol
Date Submitted by the Author:	24-Mar-2022
Complete List of Authors:	Pearson, Mark; Hull York Medical School, Wolfson Palliative Care Research Centre<br>Jackson, Gillian; Hull York Medical School, Wolfson Palliative Care Research Centre<br>Jackson, Catriona; St James's University Hospital<br>Boland, Jason; Hull York Medical School,<br>Featherstone, Imogen; University of York, Department of Health Sciences<br>Huang, Chao; University of Hull<br>Ogden , Margaret; University of Stirling, Faculty of Social Sciences<br>Sartain, Kathryn; University of Hull; York and Scarborough Teaching Hospitals NHS Foundation Trust<br>Siddiqi, Najma; University of York, Psychiatry, Hull York Medical School, York and Bradford District Care NHS Foundation Trust, Bradford, UK; Research<br>Twiddy, Maureen; University of Hull<br>Johnson, Miriam; The University of Hull, Hull York Medical School
**Primary Subject Heading**:	Palliative care
Secondary Subject Heading:	Evidence based practice, Geriatric medicine
Keywords:	PALLIATIVE CARE, Delirium & cognitive disorders < PSYCHIATRY, Protocols & guidelines < HEALTH SERVICES ADMINISTRATION & MANAGEMENT

SCHOLARONE™
Manuscripts

Improving the Detection, Assessment, Management, and Prevention of Delirium in Hospices (the DAMPen-D study): protocol for a co-design and feasibility study of a flexible and scalable implementation strategy to deliver guideline-adherent delirium care

Pearson, M.*[1], Jackson, G.[1], Jackson, C.[1,2], Boland, J.[1], Featherstone, I.[3], Huang, C.[4], Ogden, M.[5], Sartain, K.[6], Siddiqi, N.[3], Twiddy, M.[4] & Johnson, M.J[1]

*Corresponding author: Dr Mark Pearson, Wolfson Palliative Care Research Centre, Hull York Medical School, Allam Medical Building, University of Hull, Hull, HU6 7RX, UK. Tel.: +44 1482 463335

[1] Wolfson Palliative Care Research Centre, Hull York Medical School, University of Hull, Hull, HU6 7RX, UK

[2] Leeds Teaching Hospitals NHS Trust, St James's University Hospital, Leeds, LS9 7TF, UK

[3] Department of Health Sciences, University of York, York, YO10 5DD, UK

[4] Institute of Clinical & Applied Health Research, Hull York Medical School, University of Hull, Hull, HU6 7RX, UK

[5] Public involvement member

[6] York and Scarborough Teaching Hospitals NHS Foundation Trust, York Hospital, Wigginton Road, York, YO31 8HE, UK

Author email addresses:
Pearson, M. mark.pearson@hyms.ac.uk
Jackson, G. gillian.jackson@hyms.ac.uk
Jackson, C. catriona.jackson@doctors.org.uk
Boland, J. jason.boland@hyms.ac.uk
Featherstone, I. imogen.featherstone@york.ac.uk
Huang, C. chao.huang@hyms.ac.uk
Ogden, M. margaretogden@hotmail.com
Sartain, K. kathryn.sartain@york.nhs.uk
Siddiqi, N. najma.siddiqi@york.ac.uk
Twiddy, M. maureen.twiddy@hyms.ac.uk
Johnson, M.J miriam.johnson@hyms.ac.uk

Word count (excluding title page, abstract, references, figures and tables): 4008

For peer review only

Abstract

Introduction Delirium is a complex condition in which altered mental state and cognition causes severe distress and poor clinical outcomes for patients and families, anxiety and stress for the health professionals and support staff providing care, and higher care costs. Hospice patients are at high risk of developing delirium, but there is significant variation in care delivery. The primary objective of this study is to demonstrate the feasibility of an implementation strategy (designed to help deliver good practice delirium guidelines), participant recruitment, and data collection.

Methods and analysis Three work packages in three hospices in the United Kingdom with public involvement in co-design, study management and stakeholder groups: (1) experience-based co-design to adapt an existing theoretically-informed implementation strategy (Creating Learning Environments for Compassionate Care [CLECC]) to implement delirium guidelines in hospices; (2) feasibility study to explore ability to collect demographic, diagnostic, and delirium management data from clinical records (n=300), explanatory process data (number of staff engaged in CLECC activities, and reasons for non-engagement), and cost data (staff and volunteer hours and pay-grades engaged in implementation activities); and (3) realist process evaluation to assess the acceptability and flexibility of the implementation strategy (pre- and post-implementation surveys with hospice staff and management, n=30 at each time-point; interviews with hospice staff and management, n=15). Descriptive statistics, rapid thematic analysis, and a realist logic of analysis will be used be used to analyse quantitative and qualitative data, as appropriate.

Ethics and dissemination Ethical approval obtained: Hull York Medical School Ethics Committee (Ref 21/23), Health Research Authority Research Ethics Committee Wales REC7 (Ref 21/WA/0180) and Health Research Authority Confidentiality Advisory Group (Ref 21/CAG/0071). Written informed consent will be obtained from interview participants. A results paper will be submitted to an open access peer-reviewed journal and a lay summary shared with study site staff and stakeholders.

Study registration: ISRCTN55416525.

Keywords: Delirium; palliative care; guideline implementation; co-design; feasibility; realist process evaluation

Strengths and limitations of this study

- Innovative collaborative adaptation of a theoretically-informed implementation strategy (CLECC) to deliver guideline-adherent delirium care in hospices (CLECC-Pal), including evaluation of feasibility and acceptability of an implementation strategy before testing at scale.
- Research waste minimised and patient/carer burden eliminated through use of existing patient outcome and process data.
- Involvement of public members since study inception and throughout study delivery and management.
- Whilst the study hospices have diverse characteristics (locations, level of socio-economic deprivation, forms of governance), they are all drawn from a single region of the United Kingdom.

For peer review only

- The sample size for surveys and interviews may limit the extent to which the complexity of staff and management characteristics, views and experiences can be explored.

For peer review only

INTRODUCTION

Delirium is a complex condition characterised by fluctuating impairment of awareness, attention, and cognition.(1) Delirium causes severe distress for patients and families(2), anxiety and stress for the health professionals and support staff providing care,(3) poor clinical outcomes,(4, 5) and higher care costs (e.g. longer inpatient stays).(6, 7) People nearing the end of life have a high risk of delirium,(2) with risk factors such as medication, metabolic disturbance, pain, poor sleep, infection and dehydration acting cumulatively.(8) Effective delirium care is driven by prevention where possible, timely detection and non-pharmacological management, with pharmacological interventions if appropriate.(9, 10) Hospices are an important but under-researched setting for the prevention and management of delirium.

An international systematic review reported that one-third of people in adult palliative care settings had delirium on admission, with two-thirds developing delirium during the admission.(8) Across health services the health economic impact of delirium is significant. Although data are not available from palliative care settings, other estimates of health service costs from delirium show comparable costs to falls, diabetes and cardiovascular diseases.(11)

NICE Clinical Guideline 103(12) and other international guidelines(13) and standards,(14) recommend strategies for delirium assessment, prevention and management. However, this is difficult in practice, with a disconnection between improved levels of delirium knowledge and the capacity of palliative care practitioners to implement changes. A recent international qualitative systematic review identified that practical and emotional support were needed to enable staff to assess, prevent and manage delirium.(15)

A recent survey of palliative care doctors (n=335) in the United Kingdom found that 38% never used delirium guidelines and that only 13% of palliative care teams used a tool (rather than clinical judgement) to assess for delirium at first inpatient assessment, with even fewer (9%) using a tool on an ongoing basis.(16) Our survey of UK specialist palliative care units (n=220, mostly nurses)(17) found that only 10% ever used a delirium screening tool, with only 5% following NICE guidelines by screening on admission, and only 6% screening daily thereafter. The importance of delirium care has been recognised in a national survey of dying patients, with 92% rating 'being mentally aware' as "very important" and nearly as many (89%) citing 'not being a burden on family'.(18)

Delirium detection, assessment, management and prevention is complex, depending on practical support (screening tools and clinical pathways) and communication (3, 19) between family and friends, volunteers, healthcare assistants (HCAs), nurses, allied health professionals (AHPs), social workers, doctors, hospice managers and board members. It also takes place at some of the most sensitive and emotionally-fraught times in the lives of patients and their families. Therefore, guideline implementation requires a relevant and flexible strategy based on an understanding of how adaptation for different settings can be attained whilst retaining effectiveness.

To address this gap in knowledge about how to implement guideline-adherent delirium care, we shall first adapt an existing theoretically-informed implementation strategy that has been tested in acute hospital wards (*Creating Learning Environments for Compassionate Care (CLECC)*). CLECC has been found to foster and legitimise the reflection, learning, mutual support and innovation that can enable team members to progress from knowing to doing.(20) It comprises a team study day, ward manager action learning sets, peer observations of practice, and involvement of all staff in mid-shift 'cluster discussions' and

For peer review only

twice-weekly reflective discussions,(21) and is shown mapped to the TIDieR checklist(22) in Table 1. We will then test the feasibility of a subsequent quasi-experimental study to evaluate the effect of the adapted CLECC (the intervention) on hospice staff delivery of guideline-adherent delirium care and subsequent improvement in patient outcomes (reduction in the number of delirium days, with a delirium day being one where the patient was classed as having delirium using Inouye et al's chart-based instrument(23)).

Aims and objectives

This study will address key uncertainties about the implementation of guideline-adherent delirium care in hospices by demonstrating if it is possible to:

- Co-adapt an implementation strategy (Creating Learning Environments for Compassionate Care (CLECC)) for use in hospices (Work Package 1).
- Systematically and reliably collect data (including delirium diagnosis) from clinical records in a way that minimises burden for patients, families, and staff (Work Package 2).
- Collect measures of staff engagement with the implementation strategy, delivery of guideline-adherent delirium care, and the costs of staff involvement (Work Package 2).
- Collect explanatory process data about staff use of the implementation strategy (Work Package 3).
- Estimate the number of hospice sites and in-patient episodes needed for the planned national quasi-experimental study.

Work Package 1 commenced June 2021, with Work Packages 2 and 3 (and data collection) commencing August 2021. The study will be completed in February 2023.

METHODS AND ANALYSIS

Design summary

Table 2 presents the research questions and summarises the three Work Packages (WPs) that will enable the above aims and objectives to be met. Figure 1 shows the study timeline and how the work packages are inter-related.

Settings

Three adult hospices in northern England (United Kingdom). Two hospices in this study are located in socio-economically deprived urban areas (one with a significant minority ethnic group population) and one hospice in an affluent rural/urban area. One hospice is run by a national charity, with the other two hospices run by independent charities.

Patient and Public Involvement

This study supports the involvement of patient and public involvement (PPI) in accordance with the framework for good public involvement as detailed by the UK standards for public involvement.(24) Public involvement group members contributed to study design, with one member joining the monthly Study Management Group meetings, co-facilitating workshops (Work Package 1) and a further member Chairing the Study Steering Committee. The study's Public Involvement Group will meet three to four times over the duration of the study to discuss public involvement challenges in the research, the implications of emerging study

For peer review only

findings, and the development of public-facing research outputs and the next steps in the research cycle.

For peer review only

Table 1. CLECC(21) components mapped using TIDieR checklist(22)

Component	Why	What	Who	How	Where	When/How much	Tailoring & modifications	Fidelity
**Study day**	Prepare staff for the workplace elements of the intervention	**Procedure:** Introduction to CLECC Activities/discussion Questionnaires Film handouts **Materials:** PowerPoint presentation. Record of attendance. Summary of CLECC leaflet	Appointed hospice lead clinician	Classroom based to include all hospice staff	Comfortable classroom that is geographically separate from the workplace	One day at beginning of implementation period, but may require more than one study day to ensure maximum attendance	Pending Work Package 1 co-design workshops	Attendance and feedback data from hospice lead clinician.
**Action Learning sets**	Real problems from own practice and devise action plan to address	**Procedure:** Session 1: relationships & rules Session 2: valuing staff Session 3: enhancing capacity CLECC Session 4: influencing seniors	Experienced facilitator and 4-8 leads of comparable position	Face to face at hospice site	At hospice site	4 x4 hours action learning sets throughout intervention period	Pending Work Package 1 co-design workshops	Fidelity/ attendance
**Peer review**	Appreciate practice from observer perspective	**Procedure:** 2-3 x 1 hour observations Reflective summary **Materials:** Training video Poster of findings	2 team members nominate or nominated by lead and training given.	Outside of normal role to do this activity	At hospice site	Approximately 30 minute training video prior to commencing 2-3 x 1 hour observations throughout implementation	Pending Work Package 1 co-design workshops	Fidelity
**Mid-shift cluster discussions**	Opportunities for feedback, group problem solving and support to individual team members.	**Procedure:** Mid-shift 5 minute discussion	All team members on shift.	Mid-way through every shift.	At hospice site	5 minute discussion mid-shift, initially instigated by lead but then to be maintained by staff	Pending Work Package 1 co-design workshops	Fidelity
**Reflective discussions**	To prompt personal reflections and narratives about individual experiences	**Procedure:** Scheduled meetings or drop in sessions with planned activities **Materials:** Devise a sustainability plan	All team members, including senior staff and temporary staff.	Can be scheduled time during shift or drop-in sessions.	At hospice site, in a comfortable room on or near place of care.	Number of sessions dependent on the number of subjects needed to be discussed	Pending Work Package 1 co-design workshops	Fidelity

For peer review only - http://bmjopen.bmj.com/site/about/guidelines.xhtml

Table 2. Overview of study design

Work Package objective	Research question	Study type	Data collection	Timepoints
1. Refine CLECC-Pal implementation strategy	What are the core and adaptable components of an implementation strategy for guideline-adherent delirium care in hospices?	Experience-based co-design	Workshops	Before and during implementation
2. Demonstrate feasibility of future quasi-experimental study	Is it feasible to collect sufficient outcome data (both implementation and clinical), explanatory process data, and cost data in a future effectiveness evaluative study in palliative care settings?	Feasibility study	Patient demographics and delirium diagnosis & management (clinical records)<br><br>Number of staff engaged in CLECC-Pal	Baseline & follow-up<br><br>During implementation & follow-up
3. Assess acceptability and flexibility of CLECC-Pal implementation strategy	How can a co-designed implementation strategy for guideline-adherent delirium care be operationalised with fidelity to function in different hospice inpatient settings?	Realist process evaluation	Survey<br><br>Fidelity to CLECC-Pal<br><br>Interviews	Baseline & follow-up<br><br>Start, middle & end of 3-month period using CLECC-Pal<br><br>Follow-up

For peer review only

[Insert Figure 1 about here]

Work Package 1: Adaptation (Co-Design) of CLECC for guideline-adherent delirium care

An experience-based co-design (EBCD) group(25-27) of people with lived experience of delirium (themselves or in a family member or friend), staff and management from across the study sites and the region will meet for online workshops (maximum three hours duration) at months 2, 8, and 14 to adapt the CLECC strategy for use in hospices (see Figure 1). The first of these co-design workshops will be held separately for public and staff to facilitate reflection within a broader public or staff 'group' and to underpin interactions between public and staff at subsequent joint workshops. The interactions in these joint co-design workshops are considered essential for participants to share their experiences, develop an appreciation of others' experiences, and open up new ways of thinking about how to meet challenges that will directly inform co-design.(28) Consistent with the INVOLVE principles for co-producing research,(29) workshops will be co-developed with our Public Involvement group and co-facilitated by an experienced Public Involvement group member.

Potential *public* participants will be invited through existing national PPI networks to join the co-design workshops. Potential *hospice staff and management* participants (clinicians, volunteers, managers, and board members) will be invited through existing communication channels at each site and in consultation with managers. Information will be provided for potential participants with an opportunity to discuss in more detail prior to taking part. Workshops will be scheduled to fit with existing commitments and day-to-day practice at each hospice. PPI team member (MO) will provide input into all aspects of invitations, information provision, and workshop design.

We shall endeavour to maximise diversity within the workshops but acknowledge the tension between attaining diversity across every potential aspect and a maximum workable number of workshop participants of around 15. We shall keep this under review with PPI team member MO.

Central to the conduct of the workshops will be the use of 'touch points' to communicate other peoples' experiences and provide a focus to spark discussion and exploration from different perspectives.(26) Touch points are the events which significantly shape people's positive or negative experience of an event or service. It could be the sharing of a personal or professional experience of delirium care by a workshop participant, or a short film or news item about palliative care services generally or delirium specifically. These will be used to trigger discussion about the detection, assessment, prevention, and management of delirium, how CLECC can be adapted for hospices and support implementation of delirium guidelines.

Table 3 provides an overview of the schedule and content of the co-design workshops.

For peer review only - http://bmjopen.bmj.com/site/about/guidelines.xhtml

Table 3. Co-design workshops schedule and content

Workshop focus	Participants	When, duration	Content
1a. Introduction and initial refinement of CLECC-Pal	Public members	Month 2, 2 hours	• Introductions<br>• Discussion about the principles of equitable participation<br>• Discussion about the co-design approach to workshops<br>• Introduction to the CLECC strategy and exploration of priority aspects for adaptation<br>• Identification of individual working groups' role in exploring and refining site- or issue-specific aspects of the CLECC strategy before Workshop 2<br>• Agreement on feedback processes outside of the workshops and focus of agenda for Workshop 2
1b. Introduction and initial refinement of CLECC-Pal	Hospice staff and volunteers	Month 2, 2 hours	As for Workshop 1a
2. Refinement of CLECC-Pal	Public members, hospice staff and volunteers	Month 8, 3 hours	• Feedback from individual working groups<br>• Discussion of emerging findings from Work Package 3 (realist process evaluation)<br>• Specification of suggested adaptations to CLECC,<br>• Identification of further individual working groups to refine site- or issue-specific aspects of the CLECC strategy<br>• Agreement on focus of agenda for Workshop 3
3. Final specification of CLECC-Pal and celebration	Public members, hospice staff and volunteers	Month 14, 3 hours	• Feedback from individual working groups<br>• Discussion of further findings from Work Package 3 (realist process evaluation)<br>• Final specification of adaptations to CLECC<br>• Celebration of co-design outputs

For peer review only

Work package 2: Feasibility study

Feasibility will be assessed in the following key areas:

- Patients:
    - Ability to collect high quality, anonymised delirium outcome and process (extent of guideline-adherent care) data from clinical records
    - Variability of baseline delirium day measurement to calculate the sample size for a subsequent national study.
- Staff and volunteers: Number of relevant hospice staff and volunteers' participation in CLECC-Pal activities (proportion of relevant staff engaging and maintaining engagement)
- Economic: Ability to collect cost data in relation to CLECC-Pal staff activities

The co-designed CLECC-Pal (for initial version, see Table 1) will be introduced to clinical and support staff, volunteers, and managers at each hospice in a study day that will include training in guideline-recommended delirium care. The study team will support the identified clinical lead to introduce and use CLECC-Pal, including action learning sets, mid-shift 'cluster discussions', twice-weekly reflective discussions and peer observations of practice, over a minimum 12-week period. The study day ethos will emphasise how hospices should take ownership of using CLECC-Pal with only modest support from the study team.

Data collection and analysis

Patients:

Baseline and follow-up (pre and post) clinical record data will be collected. Data will be collected through remote access to the clinical record where electronic records allow, or from the paper record. At each of the three hospices, case note collection (total n=300) will comprise:

- Baseline (pre): 50 consecutive patients who completed their in-patient stay immediately prior to the start of the hospice using CLECC-Pal.
- Follow-up (post): 50 consecutive patients completing their in-patient stay from week 4 of starting use of CLECC-Pal.

Clinical record data collected by the researcher will be anonymised at the point of extraction and include:

- Demographic data (baseline only): age, sex, main medical condition, ethnicity, post code (converted to IMD score)
- Delirium diagnosis using the Inouye et al case note tool(23)
- Delirium management: including evidence of use of delirium screening tools, risk assessments and individualised delirium management care plans

Clinical record data will be extracted using an expanded version of the prospectively validated (74% sensitivity, 83% specificity) chart-based instrument developed by Inouye et al. for detecting potential delirium diagnoses from clinical records.(23) The instrument (data extraction pro-forma, see online supplemental file 1) will enable us to assess whether case-note recorded symptoms of delirium (and therefore number of patient days with delirium) can be linked to time-points during the person's admission when actions around delirium assessment, management and prevention (consistent with guidelines) did or did not take place. Our 'expanded' version of the instrument will include questions about other actions to support delirium assessment, management and prevention that may be recorded in the notes, as shown in Table 4. We shall report the percentage of clinical records where information about each of these actions is recorded. Where a person experiences multiple

episodes of delirium within one admission, each episode will be recorded separately and linked through the anonymised case number.

Where judgements about what to record on the pro-forma need to be made, justification for these will be recorded on the form. Any uncertainty about how the information in the case-notes should be recorded on the pro-forma will be discussed with a second clinician (CJ) and justification for the final decision recorded.

Table 4. Additional delirium assessment items to be derived from clinical records and means of assessing feasibility of data collection

Delirium-related action	Assessment of feasibility
Use of Richmond Agitation-Sedation Scale and 4AT screening tools	% completed<br>% re-assessments completed at appropriate timepoints
Medication reviews (to minimise deliriogenic medication)	% completed<br>% re-assessments completed at appropriate timepoints
DSM-V delirium assessment	% completed<br>% re-assessments completed at appropriate timepoints
Degree of sedation or agitation	% completed
Individualised delirium care plans	% completed<br>% reviewed at appropriate timepoints
Presence/absence of delirium	% documenting start and end of delirium episode(s)<br>% documenting delirium-free days

The number of patient records from which it was possible to extract clinical record data *longitudinally* over the duration of their inpatient admission will be reported both as a simple count and as a percentage of the total number of in-patients with a diagnosis of delirium in each hospice each month.

Sample size: Based on our pilot work in one hospice (comparable in size to the hospices in this study) which identified a monthly occurrence of 32 in-patient episodes of delirium, retrospectively collecting clinical record data for *all* patients whose episode of in-patient care is completed (up to a maximum of 50 per hospice) will provide us with enough data to answer feasibility questions about data quality and enable us to capture frequent events regarding care planning. We do not propose to investigate less-frequent events such as antipsychotic use.

Analysis: Baseline demographic and clinical characteristics of the study population (age, sex, primary medical condition, ethnicity, post code (to derive IMD)) will be presented using descriptive statistics. Mean (SD) will be reported for continuous data and raw count (number, percentage) will be reported for nominal data. The variation around baseline delirium days will be calculated to inform the sample size and number of hospices needed for the subsequent national study.

Staff and volunteers:

In consultation with operational and clinical management at each site, a hospice study lead has been identified through whom the following denominators will be established:

- Number of staff working on or rotating through the in-patient unit of the hospice
- Number of volunteers active within the in-patient unit of the hospice

For peer review only

- Total number of documented in-patient delirium episodes *or* (if total number cannot be established) number of patients with at least one case-note diagnosis of delirium per in-patient admission in the hospice

Level of staff engagement with CLECC-Pal during the implementation period will be assessed weekly by the hospice study lead completing a rapid report of numbers of:

- staff *indirectly* involved in delivering delirium care who attend the team study day, action learning sets, feedback following peer observations of practice, mid-shift cluster discussions, and reflective discussions
- staff and volunteers who do not engage with CLECC-Pal
- staff and volunteers who decrease or stop their engagement with CLECC-Pal
- peer observations of practice achieved
- people approached, reported by professional group and role, who agree to participate in using CLECC-Pal

The rapid report will also record reasons for:

- staff and volunteers' non-engagement or dropout
- modifications made in the use of CLECC-Pal

Quantitative data will be analysed descriptively using radar plots. Qualitative data will be rapidly analysed deductively using a Framework approach.(30) Analyses will inform more detailed exploration in interviews (WP3) and will be shared with participating hospices to inform their ongoing use of CLECC-Pal.

Economic:

We will assess the feasibility of collecting data about the costs of using CLECC-Pal:

- Number of hours spent by members of staff and volunteers in CLECC-Pal activities, linked to pay-grade where possible

Work Package 3: Realist Process Evaluation

Critiques of process evaluations have highlighted the importance of methods that can use theory to explore how contexts and mechanisms interact,(31-33) as recognised in the revised Medical Research Council framework.(34) We shall use realist evaluation(35) to capture staff and management insights into how individual-, team-, and organisational-level contexts affect these interactions during implementation,(36) refining Normalisation Process Theory's (NPT) propositions about the mechanisms of coherence, cognitive participation, collective action, and reflexive monitoring.(37-39). Definitions of realist terms are shown in Table 5. This theoretically-informed understanding of how the implementation strategy *functions*(40) will enable us to explain how hospices may operationalise CLECC-Pal in different ways to achieve the same desired outcomes (for example, by running online learning rather than a team study day, or using self-reflection on practice rather than peer observation).

Table 5. Definition of realist terms used in Work Package 3

Term	Definition
Context	Individual, team, organisational, or other factors that enable or constrain the operation of mechanisms.(41) This includes social phenomena such as rules, norms and values, meaning that contexts are not straightforwardly analogous with settings.(42)

For peer review only - http://bmjopen.bmj.com/site/about/guidelines.xhtml

Mechanism	The interaction of a programme's resources or opportunities with individuals' or teams' reasoning.(41)
Outcome	The 'demi-regular' occurrences arising from particular configurations of contexts and mechanisms.(43) Consistent with the recognition in realist ontology of the dynamic and non-linear nature of open systems in the social world,(44) 'outcomes' may be better understood as semi-stable processes.
Programme Theory	A middle-range theoretical explanation of how (implementation) programme activities relate to underlying theory. Even if not explicitly stated, programme theories contain ideas about how best to address challenges to achieving intended goals (including how to proactively manage these challenges)(45)

Identification, sampling and consent

Surveys

All hospice staff involved in direct patient care or management, as well as those directly involved in patient care (volunteers, support staff, board members with a hospice governance role) will be eligible. Minimum sample size of 10 at each hospice (total n=30). Eligible participants will be sent a link to the anonymous survey, for which completion online will be taken as implied consent.

Interviews

A purposive sampling strategy at each site will draw from a sampling frame that includes all hospice staff involved in direct patient care or management, volunteers, support staff, and board members at each study site. Within the constraints of an exploratory sample size (five staff and volunteers, and two members of management and/or executive board at each site; minimum total n=15), we shall endeavour to maximise variation in participant characteristics and roles, prioritising sampling that will enable comparison between those who do and do *not* take part. Written informed consent will be obtained. Interviews will be conducted at a time suitable for participants and may be face-to-face or remote, according to participant preference.

Data collection and analysis

Staff and volunteers' pre- and post-implementation experiences (survey):

Survey using a modified and piloted Normalisation Measurement Instrument (NoMad).(46) of staff and volunteers' perceptions and experiences of implementation, in relation to each NPT mechanism, before and after using the CLECC-Pal implementation strategy.

Quantitative Likert scale responses will be analysed descriptively using radar plots. Free-text responses will be deductively thematically-analysed using the framework of NPT mechanisms (coherence, cognitive participation, collective action, and reflexive monitoring), allowing for inductive thematic analysis if responses do not fit within the framework. Thematic patterns and outliers will be identified. The analysis will also inform the structure, content, and focus of the staff and volunteer interviews.

Staff and volunteers' post-implementation experiences (interviews):

For peer review only

Realist interviews are distinct from conventional qualitative semi-structured interviews as they adopt a ‘teacher-learner’ approach. This involves presenting theory to participants so that they can communicate their own experiences and views that may refute, refine, or expand the theory.(47) In practice, the realist interviewer presents theory (context-mechanism-outcome configurations) in a form comprehensible to the participant and follows-up flexibly with further questions tailored to the participant’s understanding, to ensure that the discussion enables theory-refinement rather than simply a discussion of experiences. Interviews will build on Murray et al’s.(48) operationalisation of NPT for the development and optimisation of interventions within trials (see Table 6).

Interview topics will include, but not be limited to, experiences of CLECC-Pal’s acceptability and fit, rationale for any modifications to CLECC-Pal, perceived changes in communication between those caring for patients at-risk of delirium, changes in care practices, perceptions about how CLECC-Pal is achieving (or not) the intended effects and, if appropriate, how these impacts could be sustained. Interview questions will be informed by emerging site-specific data from the co-design and feasibility work packages, as well as from the process evaluation survey. Graphical summaries of data, such as radar plots, will be used in the interviews to communicate this emerging data to participants, link to theory, and to support discussion that enables implementation theory to be refined.(47, 49) Views of study processes will also be sought. It is envisaged that interviews will last no longer than 30 minutes, but participants will be given the opportunity for a longer interview if they wish.

Interviews will be recorded and transcribed. Before commencing analysis, interview transcripts will be read and re-read to allow familiarisation with the content that will enable theory-building and refinement rather than rote coding of contexts, mechanisms and outcomes (although coding of these configurations may also play an important role in theory-building and refinement). Analysis to identify contextualised explanations of how mechanisms of implementation are understood to lead to certain outcomes will be structured using the reasoning processes identified by Pawson (juxtaposition, reconciliation, adjudication, consolidation, and situating(50)). We shall operationalise these reasoning processes using the analytic questions for building and refining programme theory identified by Pearson et al.(51)

Work Package 3 methods and findings will be reported consistent with the RAMESES reporting standards.(43)

For peer review only

Table 6. Normalisation Process Theory 'Contribution' mechanisms and their relationship to data collection in interviews

Mechanism	Definition(37)	Theoretical propositions(38)	Potential interview questions(48)
1.Coherence	Agents attribute meaning to a complex intervention and make sense of its possibilities within their field of agency. They frame how participants make sense of, and specify, their involvement in a complex intervention.	1.1 Embedding is dependent on work that defines and organises a practice as a cognitive and behavioural ensemble.<br>1.2 Embedding work is shaped by factors that promote or inhibit actors' apprehension of a practice as meaningful.<br>1.3 The production and reproduction of coherence in a practice requires that actors collectively invest meaning in it.	Is CLECC-Pal:<br>- easy to describe?<br>- clearly distinct from other strategies?<br>- have a clear purpose for all participants?<br>Do participants have a shared sense of purpose?<br>What benefits will the intervention bring and to whom?<br>Are these benefits likely to be valued by potential participants?<br>Will CLECC-Pal fit with the overall goals and activity of the organisation?
2.Cognitive Participation	Agents legitimise and enrol themselves and others into a complex intervention. They frame how participants become members of a specific community of practice.	2.1 Embedding is dependent on work that defines and organises the actors implicated in a practice.<br>2.2 Embedding work is shaped by factors that promote or inhibit actors' participation.<br>2.3 The production and reproduction of a practice requires that actors collectively invest commitment in it.	Are target user groups likely to think that CLECC-Pal is a good idea?<br>Will they see the point of CLECC-Pal?
3.Collective Action	Agents mobilise skills and resources and enact a complex intervention. They frame how participants realise and perform the intervention in practice.	3.1 Embedding is dependent on work that defines and operationalises a practice.<br>3.2 Embedding work is shaped by factors that promote or inhibit actors' enacting it.<br>3.3 The production and reproduction of a practice requires that actors collectively invest effort in it.	How will CLECC-Pal affect the work of user groups?<br>Will CLECC-Pal promote or impede their work?<br>Will staff require extensive training before they can use CLECC-Pal?<br>How compatible with existing work practices is CLECC-Pal?<br>What impact will CLECC-Pal have on division of labour, resources, power, and responsibility between different professional groups?<br>Will CLECC-Pal fit with the overall goals and activity of the organisation?

For peer review only

Mechanism	Definition(37)	Theoretical propositions(38)	Potential interview questions(48)
4.Reflexive Monitoring	Agents assemble and appraise information about the effects of a complex intervention within their field of agency, and utilise that knowledge to reconfigure social relations and action. They frame how participants collect and utilise information about the effects of the intervention.	4.1 Embedding is dependent on work that defines and organises the everyday understanding of a practice.<br>4.2 Embedding work is shaped by factors that promote or inhibit appraisal.<br>4.2 The production and reproduction of a practice requires that actors collectively invest in its understanding.	How are users likely to perceive CLECC-Pal once it has been used for a while?<br>Is CLECC-Pal likely to be perceived as advantageous for patients or staff?<br>Will it be clear what effects CLECC-Pal has had?<br>Can users contribute feedback about CLECC-Pal once it is in use?<br>Can CLECC-Pal be adapted or improved on the basis of experience?

For peer review only

Ethics and dissemination

Ethical approval for the study has been obtained from Hull York Medical School Ethics Committee (Ref.: 21/23), Health Research Authority Research Ethics Committee Wales REC7 (Ref.: 21/WA/0180) and Health Research Authority Confidentiality Advisory Group (Ref.: 21/CAG/0071). Confidentiality Advisory Group approval allows the study researcher access to the clinical records to extract data without patient consent. The study is publicised in the hospices during the data collection period and patients/representatives may opt out if they do not wish their data to be used. Written informed consent will be obtained from interview participants.

he primary objective of this study is to inform a future quasi-experimental multi-site comparative evaluation. We shall do this by demonstrating the feasibility (or otherwise) of the implementation strategy ('intervention'), participant recruitment, and data collection, in addition informing decisions about the most appropriate study design for a future multi-site comparative evaluation. However, as argued by Thabane et al.,(52) communicating findings from feasibility studies remains critically important for ensuring that resources are not spent on either duplicating the feasibility study or funding research uninformed by the findings of a relevant feasibility study. We shall therefore prepare a full report of the study's methods and findings for the funder and submit a manuscript reporting the findings to an open access peer-reviewed journal. The study's findings will also be submitted for oral presentation at one national health services research conference and one international palliative care conference. A Plain English summary of study findings will be prepared for distribution through palliative care clinical networks (including Hospice UK) and Public Involvement groups.

Discussion

This study will address key uncertainties about the implementation of guideline-adherent delirium care in hospices - the feasibility of using a theoretically-informed, co-developed implementation strategy (CLECC-Pal); collecting demographic, diagnostic, and delirium management data from clinical records; collecting measures of staff engagement; and collecting explanatory process data about staff use of CLECC-Pal. This will enable us to estimate the number of hospice sites and in-patient episodes needed for the planned national quasi-experimental study, for which we outline the design considerations below. The study has clear strengths in public involvement and in minimising research waste by using existing process and outcome data. There are also limitations in the study, for example, hospices are all drawn from a single region of the United Kingdom and the sample size for surveys and interviews may limit the extent to which the complexity of staff and management characteristics, views and experiences can be explored. Nevertheless, the study hospices have diverse characteristics (locations, level of socio-economic deprivation, forms of governance) and we shall purposively sample staff and management (for interviews) to maximise the range of professional and role characteristics.

We have developed this feasibility study to inform future decisions about evaluative study design that balances scientific rigour and practical considerations. In doing so, we first appraised an interrupted time series design that would enable naturalistic data collection, but considered this unrealistic as powering the study would likely require 12 months pre- and post-intervention data collection.(53) Second, we appraised a randomised stepped wedge design, but considered implementation research permutations of this design unlikely to be

For peer review only

feasible due to the real-world setting (if using a head-to-head rollout design) or length of time required (if using a pairwise enrolment rollout design).(54)

Consistent with current thinking in implementation research for investigators to consider quasi-experimental study designs that can assess the impact of context over time(55), we plan to work towards an evaluative study design that uses natural variation in the introduction of the implementation strategy to allow a *non-randomised* stepped wedge design (CLECC-Pal supported delirium care vs. delirium care as usual). Our audit data indicate that this would be realistic given an annual admission rate of 192-384 in the 10-20 bedded study site hospices which have a 40-60% incidence of delirium.

Whilst hospices are relatively homogeneous in terms of care delivery by health professionals (e.g. standardised national training programme for doctors, national standards for nursing practice), the wide referral base of hospices mean that in-patients tend to be heterogeneous in relation to type and stage of disease, ethnicity, socio-economic status, and so on. For the future evaluative study, we shall estimate the intraclass correlation coefficient (ICC) using pre-intervention patient outcome data (delirium-free days) from the feasibility study, thus enabling a sample size calculation powered on the primary outcome for the future evaluative study.

We are mindful of a recent systematic review of feasibility studies which identified a lack of consistency in the use of terminology, a predominance of feasibility issues relating to preparation for randomised-controlled trials, and an absence of clear guidance about when "sufficient insight about uncertainties" had been achieved for progression to an evaluation study.(56, p.10) However, we are confident in stating minimum recruitment targets for the use of CLECC-Pal (fidelity to core components) and 4AT screening tool at baseline and daily, that will be necessary for a future evaluative study to be considered feasible:

- ≥80%, proceed
- 60 – 80% with mitigating factors, proceed
- <60% not feasible

Contributors: MP and MJ led study conceptualisation and design, with contributions from CJ, JB and NS. MP and MJ led development of analysis plans, with contributions from CJ, CH and MT. MP led the writing process and drafted the original protocol with input from GJ and MJ. Critical review of the protocol and contributions to refinement to: co-design work package from GJ, MO, IF and MT; feasibility work package from GJ, CJ, JB, IF, CH, MO, KS, NS, MT and MJ; process evaluation work package from GJ, CJ, IF, MT and MJ. All authors take responsibility for the protocol and approved the final version of this paper.

Funding: This work is supported by Yorkshire Cancer Research (Award reference number HEND405DEL).

Competing interests: The authors declared no potential conflicts of interest with respect to the research, authorship, and/or publication of this article.

Acknowledgments: Creating Learning Environments for Compassionate Care (CLECC) © 2020 University of Southampton. Used under non-exclusive licence.

References:

1. American Psychiatric Association. Diagnostic and Statistical Manual of Mental Disorders, Fifth Edition (DSM-5). Arlington, VA: American Psychiatric Publisher; 2013.
2. Hosie A, Davidson PM, Agar M, Sanderson CR, Phillips J. Delirium prevalence, incidence, and implications for screening in specialist palliative care inpatient settings: A systematic review. Palliative Medicine. 2012;27:486–98.
3. Brajtman S, Higuchi K, McPherson C. Caring for patients with terminal delirium: palliative care unit and home care nurses' experiences. International Journal of Palliative Nursing. 2006;12(4):150-6.
4. Fang CK, Chen HW, Liu SI, Lin CJ, Tsai LY, Lai YL. Prevalence, detection and treatment of delirium in terminal cancer inpatients: a prospective survey. Jpn J Clin Oncol. 2008;38(1):56-63.
5. Lawlor PG, Gagnon B, Mancini IL, Pereira JL, Hanson J, Suarez-Almazor ME, et al. Occurrence, causes, and outcome of delirium in patient with advanced cancer: a prospective study. Arch Intern Med. 2000;160(6):786-94.
6. Weinrebe W, Johannsdottir E, Karaman M, Fusgen I. What does delirium cost? An economic evaluation of hyperactive delirium. Z Gerontol Geriatr. Z Gerontol Geriatr. 2016;49(1):52-8.
7. Zaubler TS, Murphy K, Rizzuto L, Santos R, Skotzko C, Giordano J, et al. Quality improvement and cost savings with multicomponent delirium interventions: replication of the Hospital Elder Life Program in a community hospital. Psychosomatics. 2013;54(3):219-26.
8. Watt CL, Momoli F, Ansari MT, Sikora L, Bush SH, Hosie A, et al. The incidence and prevalence of delirium across palliative care settings: A systematic review. Palliative Medicine. 2019;DOI: 10.1177/0269216319854944.
9. Oh ES, Fong TG, Hshieh TT, Inouye SK. Delirium in older persons: Advances in diagnosis and treatment. JAMA. 2017;318(12):1161-74.
10. Thom RP, Levy-Carrick NC, Bui M, Silbersweig D. Delirium. American Journal of Psychiatry. 2019;176(10):785-93.
11. Leslie DL, Inouye SK. The importance of delirium: economic and societal costs. J Am Geriatr Soc. 2011;59 (Suppl 2):S241-3.
12. NICE. Delirium: prevention, diagnosis and management - Clinical Guideline 103. London: National Institute for Health & Care Excellence; 2010.
13. Scottish Intercollegiate Guidelines Network. Risk reduction and management of delirium: A national clinical guideline. Edinburgh: NHS Scotland; 2019.
14. Australian Commission on Safety and Quality in Health Care. Delirium Clinical Care Standard. Sydney: ACSQHC; 2016.
15. Featherstone I, Hosie A, Siddiqi N, Grassau P, Bush SH, Taylor J, et al. The experience of delirium in palliative care settings for patients, family, clinicians and volunteers: A qualitative systematic review and thematic synthesis. Palliative Medicine. 2021;35(6):988-1004.
16. Boland JW, Kabir M, Bush SH, Spiller JA, Johnson MJ, Agar M, et al. Delirium management by palliative medicine specialists: a survey from the association for palliative medicine of Great Britain and Ireland. BMJ Supportive & Palliative Care. 2019.
17. Woodhouse R, Siddiqi N, Boland JW, Featherstone I, Johnson MJ. Screening for delirium: A survey of delirium screening practice in specialist palliative care units in the UK. BMj Supportive & Palliative Care. 2020;[Submitted for publication].
18. Steinhauser KE, Christakis NA, Clipp EC. Factors Considered Important at the End of Life by Patients, Family, Physicians, and Other Care Providers. JAMA. 2000;284(19):2476-82.
19. Hosie A, Lobb E, Agar M, Davidson PM, Phillips J. Identifying the barriers and enablers to palliative care nurses' recognition and assessment of delirium symptoms: a qualitative study. Journal of Pain & Symptom Management. 2014;48(5):815-30.
20. Bridges J, Pickering RM, Barker H, Chable R, Fuller A, Gould L, et al. Implementing the Creating Learning Environments for Compassionate Care (CLECC) programme in acute hospital settings: a pilot RCT and feasibility study. Health Serv Deliv Res. 2018;6(33).
21. Bridges J, Gould L, Libberton P, Schoonhoven L. Creating learning environments for compassionate care: a programme to support nursing leaders and teams to deliver compassionate hospital care - Manual for Practice Development Leads. Southampton: University of Southampton; 2020.
22. Hoffmann TC, Glasziou PP, Boutron I, Milne R, Perera R, Moher D, et al. Better reporting of interventions: template for intervention description and replication (TIDieR) checklist and guide. BMJ. 2014;348:g1687.

For peer review only

23. Inouye SK, Leo-Summers L, Zhang Y, Bogardus ST, Leslie DL, Agostini JV. A chart-based method for identification of delirium: validation compared with interviewer ratings using the Confusion Assessment Method. J Am Geriatr Soc. 2005;53:312-8.
24. UK Public Involvement Standards Development Partnership. UK Standards for Public Involvement. London: NIHR Central Commissioning Facility; 2019.
25. Bate P, Robert G. Experience-based design: from redesigning the system around the patient to co-designing services with the patient. Quality & safety in health care. 2006;15(5):307-10.
26. Locock L, Robert G, Boaz A, Vougioukalou S, Shuldham C, Fielden J, et al. Testing accelerated experience-based co-design: a qualitative study of using a national archive of patient experience narrative interviews to promote rapid patient-centred service improvement. Health Serv Deliv Res. 2014;2(4).
27. Pearson N, Naylor P-J, Ashe MC, Fernandez M, Yoong SL, Wolfenden L. Guidance for conducting feasibility and pilot studies for implementation trials. Pilot and Feasibility Studies. 2020;6(167).
28. Iedema R, Merrick E, Piper D, Britton K, Gray J, Verma R, et al. Codesigning as a discursive practice in emergency health services: the architecture of deliberation. Journal of Applied Behavioral Science. 2010;46(1):73-91.
29. INVOLVE. Guidance on co-producing a research project. Southampton: INVOLVE; 2018.
30. Ritchie J, Lewis J, McNaughton-Nicholls C, Ormston R, editors. Qualitative Research Practice: A guide for social science students and researchers. 2nd edition ed. London: Sage; 2013.
31. Moore GF, Audrey S, Barker M, Bond L, Bonell C, Hardeman W, et al. Process evaluation of complex interventions: Medical Research Council guidance. BMJ. 2015;350:h1258.
32. Murdoch J. Process evaluation for complex interventions in health services research: analysing context, text trajectories and disruptions. BMC Health Serv Res. 2016;16(407).
33. Wells M, Williams B, Treweek S, Coyle J, Taylor J. Intervention description is not enough: evidence from an in-depth multiple case study on the untold role and impact of context in randomised controlled trials of seven complex interventions. Trials. 2012;13:95-111.
34. Skivington K, Matthews L, Simpson SA, Craig P, Baird J, Blazeby JM, et al. A new framework for developing and evaluating complex interventions: update of Medical Research Council guidance. BMJ. 2021;374:n2061.
35. Pawson R. The Science of Evaluation: A Realist Manifesto. London: Sage; 2013.
36. O'Brien R, Buston K, Wight D, McGee E, White J, Henderson M. A realist process evaluation of Enhanced Triple P for Baby and Mellow Bumps, within a Trial of Healthy Relationship Initiatives for the Very Early years (THRIVE): study protocol for a randomized controlled trial. Trials. 2019;20(1):351.
37. May C. Towards a general theory of implementation. Implementation science : IS. 2013;8:18.
38. May C, Finch T. Implementing, Embedding, and Integrating Practices: An Outline of Normalization Process Theory. Sociology. 2009;43(3):535-54.
39. May CR, Cummings A, Girling M, Bracher M, Mair FS, May CM, et al. Using Normalization Process Theory in feasibility studies and process evaluations of complex healthcare interventions: a systematic review. Implementation science : IS. 2018;13(1):80.
40. Hawe P, Shiell A, Riley T. Theorising interventions as events in systems. American journal of community psychology. 2009;43(3-4):267-76.
41. Pearson M, Chilton R, Wyatt K, Abraham C, Ford T, Woods HB, et al. Implementing health promotion programmes in schools: a realist systematic review of research and experience in the United Kingdom. Implementation science : IS. 2015;10(1):149.
42. Pawson R, Tilley N. Realistic Evaluation. London: Sage Publications; 1997.
43. Wong G, Westhorp G, Manzano A, Greenhalgh J, Jagosh J, Greenhalgh T. RAMESES II reporting standards for realist evaluations. BMC medicine. 2016;14(1):96.
44. Sayer A. Realism and Social Science. London: Sage; 2000.
45. Pawson R, Owen L, Wong G. The Today Programme's contribution to Evidence-Based Policy. Evaluation. 2010;16(2):211-3.
46. Finch TL, Girling M, May CR, Mair FS, Murray E, Treweek S, et al. Nomad: Implementation measure based on Normalization Process Theory. [Measurement instrument]. 2015 [01/12/2021]. Available from: http://www.normalizationprocess.org.
47. Manzano A. The craft of interviewing in realist evaluation. Evaluation. 2016;22(3):342-60.
48. Murray E, Treweek S, Pope C, MacFarlane A, Ballini L, Dowrick C, et al. Normalisation process theory: a framework for developing, evaluating and implementing complex interventions. BMC medicine. 2010;8(63).

For peer review only

49. Mukumbang FC, Marchal B, Van Belle S, van Wyk B. Using the realist interview approach to maintain theoretical awareness in realist studies. Qualitative Research. 2019:146879411988198.
50. Pawson R. Evidence-Based Policy: A Realist Perspective. London: Sage Publications; 2006.
51. Pearson M, Brand SL, Quinn C, Shaw J, Maguire M, Michie S, et al. Using realist review to inform intervention development: methodological illustration and conceptual platform for collaborative care in offender mental health. Implementation science : IS. 2015;10(1):134.
52. Thabane L, Ma J, Chu R, Cheng J, Ismaila A, Rios LP, et al. A tutorial on pilot studies: the what, why and how. BMC Medical Research Methodology. 2010;10(1).
53. Zhang F, Wagner AK, Ross-Degnan D. Simulation-based power calculation for designing interrupted time series analyses of health policy interventions. Journal of Clinical Epidemiology. 2011;64:1252-61.
54. Brown CH, Curran G, Palinkas LA, Aarons GA, Wells KB, Jones L, et al. An Overview of Research and Evaluation Designs for Dissemination and Implementation. Annu Rev Public Health. 2017;38:1-22.
55. Kemp CG, Wagenaar BH, Haroz EE. Expanding Hybrid Studies for Implementation Research: Intervention, Implementation Strategy, and Context. Frontiers in Public Health. 2019;7.
56. Hallingberg B, Turley R, Segrott J, Wight D, Craig P, Moore L, et al. Exploratory studies to decide whether and how to proceed with full-scale evaluations of public health interventions: a systematic review of guidance. Pilot and Feasibility Studies. 2018;4(104).

Figures:

Figure 1. Study flowchart and timeline summary

For peer review only

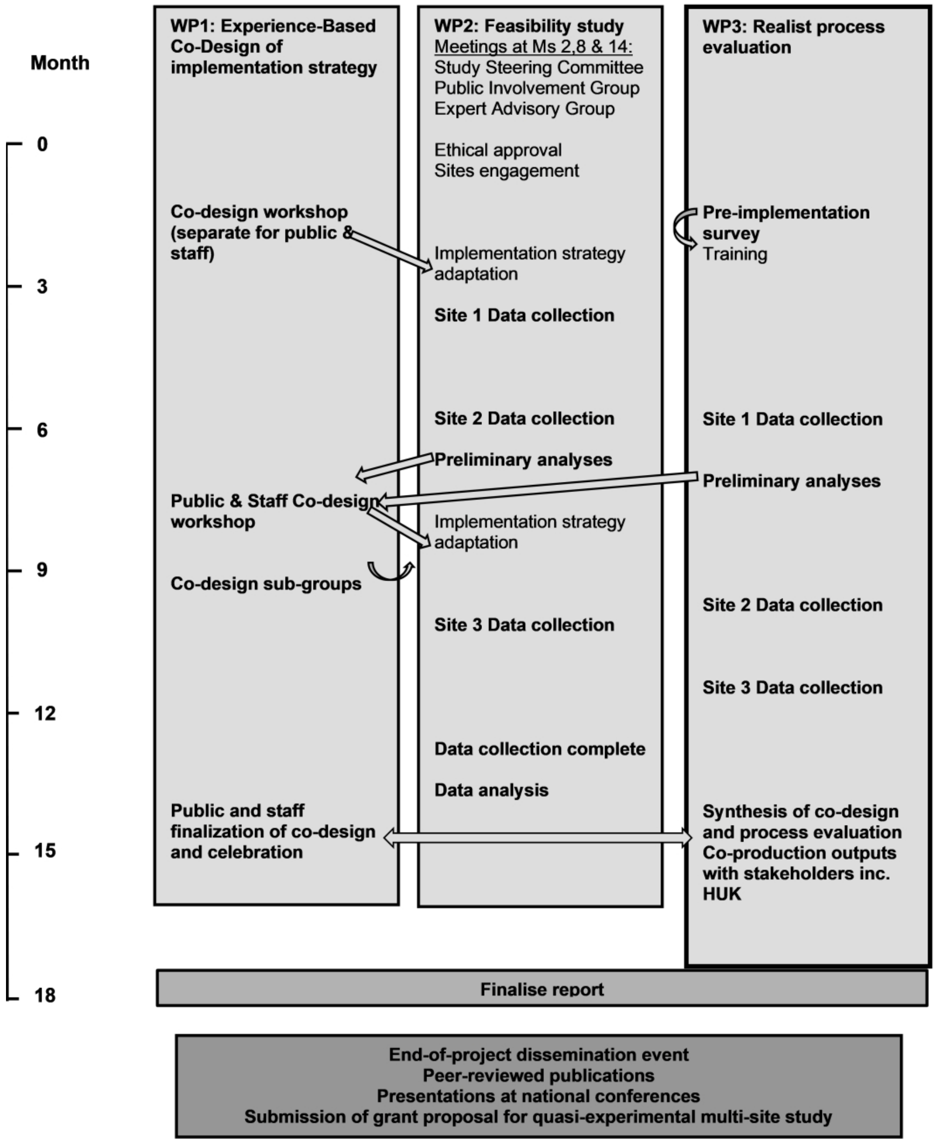

Figure 1 Study flowchart and timeline summary

1034x1326mm (96 x 96 DPI)

Online supplemental file 1
'Improving the Detection, Assessment, Management, and Prevention of Delirium in Hospices (the DAMPen-D study): Protocol for a co-design and feasibility study of a flexible and scalable implementation strategy to deliver guideline-adherent delirium care.'

**Case Number_Pre**	Non-identifiable ID number	N/A	Alphnumeric
**Age_Pre**	Patient age	Integer	Years
**Sex_Pre**	Patient gender	Categorical	Male, female, other
**Diagnosis_Pre**	Patient diagnosis	String	Cancer etc.
**Ethnicity_pre**	Patient ethnicity	Categorical	Black, white etc.
**IMD score_Pre**	Postcode converted	Float	
**Adm_Ac_conf_state_Pre**	Evidence of acute confusional state on admission	Binary	Yes/No
**Adm_screen_Pre**	Patient screened for delirium on admission	Binary	Yes/No
**Adm_Screen_by_Pre**	If screened, who completed screening	Categorical	Doctor, Nurse practitioner, Registered Nurse, Other (specify)
**Adm_screen_type_Pre**	If screened, name of screening tool	Alphanumeri c	4AT etc.
**Adm_screen_result_Pre**	If screened, result	Binary	Positive/Negative
**Adm_no_screen_just-Pre**	Was justification given for not screening	Binary	Yes/No
**Adm_no-screen_just-verbatim-Pre**	Was justification given for not screening	String	Verbatim text

For peer review only - http://bmjopen.bmj.com/site/about/guidelines.xhtml

Online supplemental file 1
'Improving the Detection, Assessment, Management, and Prevention of Delirium in Hospices (the DAMPen-D study): Protocol for a co-design and feasibility study of a flexible and scalable implementation strategy to deliver guideline-adherent delirium care.'

**Adm_risk-ass-Pre**	If screening negative or not done was risk assessment carried out?	Binary	Yes/No
**Adm-risk-ass_result_Pre**	If risk assessment carried completed results	Binary	Positive/Negative
**Adm_prev_meas_Pre**	If risk assessment positive were preventive measures put in place	Binary	Yes/No
**Adm_Judge_rationale_pre**	If researcher judgement was required for any of above, give rationale	String	Free text
**Dur_adm_Ac_conf_state_pre**	Evidence of acute confusional state during admission	Binary	Yes/No
**Dur_adm_ Multi_ep_cog_dys_Pre**	Multiple episodes of cognitive dysfunction during admission	Binary	Yes/No
**Dur_adm_ Multi_ep_cog_dys_no_Pre**	Multiple episodes of cognitive dysfunction during admission	Integer	1,2,3

For peer review only - http://bmjopen.bmj.com/site/about/guidelines.xhtml

Online supplemental file 1
'Improving the Detection, Assessment, Management, and Prevention of Delirium in Hospices (the DAMPen-D study): Protocol for a co-design and feasibility study of a flexible and scalable implementation strategy to deliver guideline-adherent delirium care.'

**Dur_adm_screen_Pre**	If patient had multiple episodes was the patient screened	Binary	Yes/No
**Dur_adm_screen_type_Pre**	If screened, name of tool	Alphanumeri c	4AT etc.
**Dur_Adm_Screen_result_Pre**	Result of screening during admission	Binary	Positive/negative
**Dur_adm_Sceen_by_Pre**	Who completed screening during admission	Categorical	Doctor, Nurse practitioner, Registered Nurse, Other (specify)
**Dur_adm_Judge_rationale_Pre**	If researcher judgement was required for any of above, give rationale	String	Verbatim text
**Case_rec_ac_conf_reported_by_Pr e**	Who reported the first episode of acute confusion in the case record	Categorical	Doctor, Nurse practitioner, Registered Nurse, Other (specify)
**Case_rec_date_first_ep_Pre**	Date of first episode of acute confusion in the case record	Date	10.10.2021
**Case_rec_time_first_ep_Pre**	Time of first episode of acute confusion in case record	Time	24hr format

For peer review only - http://bmjopen.bmj.com/site/about/guidelines.xhtml

Online supplemental file 1
'Improving the Detection, Assessment, Management, and Prevention of Delirium in Hospices (the DAMPen-D study): Protocol for a co-design and feasibility study of a flexible and scalable implementation strategy to deliver guideline-adherent delirium care.'

**Case_rec_verbatim_ref_ac_conf_Pre**	Describe each reference to acute confusion in the case record	String	Verbatim text
**Case_rec_ac_conf_tot_days_Pre**	Total duration of acute confusion in days as determined by all the references in the case record	Integer	5 (days) or 0 days if none
**Case_rec_Improve_revers_Pre**	Any evidence of improvement or reversibility of acute confusion during the stay	Categorical	Yes/No/Unsure
**Case_rec_ev_descr_pre**	Describe evidence of reversibility	String	Free text
**Case_rec_Del_present_Pre**	Delirium present	Categorical	Yes/No
**Case_rec_subtype_Pre**	If delirium present what subtype	Categorical	Hypo/Hyper/Mixed
**Case_rec_del_med_ass_Pre**	Medical assessment (DSM-V delirium assessment) to assess for delirium	Binary	Yes/No
**Case_rec_diag_doc_Pre**	Diagnosis of delirium recorded	Categorical	Yes/No

For peer review only - http://bmjopen.bmj.com/site/about/guidelines.xhtml

Online supplemental file 1
'Improving the Detection, Assessment, Management, and Prevention of Delirium in Hospices (the DAMPen-D study): Protocol for a co-design and feasibility study of a flexible and scalable implementation strategy to deliver guideline-adherent delirium care.'

**Case_rec_judge_rationale_Pre**	If researcher judgement was required for any of above, give rationale	String	Free text
**Invest_del_ ass_rev_cause_Pre**	Assessment for reversible causes of delirium	Binary	Yes/No
**Invest_med_rev_Pre**	Was a medication review conducted	Binary	Yes/No
**Invest_rev_cause_treat_Pre**	Was a treatment instigated for a reversible cause of delirium	Binary	Yes/No
**Invest_ judge_rationale_Pre**	If researcher judgement was required for any of above, give rationale	String	Free text
**Del_care_plan_Pre**	Delirium care plan documented	Binary	Yes/no
**Del_sev_Pre**	Was delirium severity assessed	Categorical	RASS-PAL + hallucination,RASS -PAL only, hallucination only, other specify, No

For peer review only - http://bmjopen.bmj.com/site/about/guidelines.xhtml

Online supplemental file 1
'Improving the Detection, Assessment, Management, and Prevention of Delirium in Hospices (the DAMPen-D study): Protocol for a co-design and feasibility study of a flexible and scalable implementation strategy to deliver guideline-adherent delirium care.'

**Harm_distress_behaviour_Pre**	Did patient display behaviours harmful or distressing to self or others	Binary	Yes/No
**Sedative_admin_during_del_Pre**	Was sedative administered during period of delirium	Binary	Yes/No
**Sedative_med_type**	Sedative medication type	String	Name of medication
**Sed_ind_Pre**	Sedative medication administered for	Categorical	Delirium, anxiety, breathlessness, nausea, terminal agitation, other, unclear
**Del_risk_discuss_patient_fam**	Was delirium risk and prevention discussed with patients and families of patients without delirium on admission	Categorical	Yes/No/unable
**Del_ep_discuss_patient_Pre**	Was episode of delirium discussed with the patient	Categorical	Yes/No/Unable

For peer review only - http://bmjopen.bmj.com/site/about/guidelines.xhtml

Online supplemental file 1
'Improving the Detection, Assessment, Management, and Prevention of Delirium in Hospices (the DAMPen-D study): Protocol for a co-design and feasibility study of a flexible and scalable implementation strategy to deliver guideline-adherent delirium care.'

**Del_ep_discuss_patient_family_Pre**	Was episode of delirium discussed with the patient's family	Categorical	Yes/No/Unable
**Del_info_Pre**	Was any written information about delirium provided to patient or family	Categorical	Yes/No/Unable

For peer review only - http://bmjopen.bmj.com/site/about/guidelines.xhtml

CONSORT 2010 checklist of information to include when reporting a pilot or feasibility trial*

Section/Topic	Item No	Checklist item	Reported on page No
**Title and abstract**			
	1a	Identification as a pilot or feasibility randomised trial in the title	1
	1b	Structured summary of pilot trial design, methods, results, and conclusions (for specific guidance see CONSORT abstract extension for pilot trials)	2
**Introduction**			
Background and objectives	2a	Scientific background and explanation of rationale for future definitive trial, and reasons for randomised pilot trial	3-4
	2b	Specific objectives or research questions for pilot trial	4, 6
**Methods**			
Trial design	3a	Description of pilot trial design (such as parallel, factorial) including allocation ratio	NA (protocol)
	3b	Important changes to methods after pilot trial commencement (such as eligibility criteria), with reasons	NA (protocol)
Participants	4a	Eligibility criteria for participants	9
	4b	Settings and locations where the data were collected	4
	4c	How participants were identified and consented	9, 12, 15
Interventions	5	The interventions for each group with sufficient details to allow replication, including how and when they were actually administered	5
Outcomes	6a	Completely defined prespecified assessments or measurements to address each pilot trial objective specified in 2b, including how and when they were assessed	9-11, 12
	6b	Any changes to pilot trial assessments or measurements after the pilot trial commenced, with reasons	NA (protocol)
	6c	If applicable, prespecified criteria used to judge whether, or how, to proceed with future definitive trial	16
Sample size	7a	Rationale for numbers in the pilot trial	9-10
	7b	When applicable, explanation of any interim analyses and stopping guidelines	NA
Randomisation:			
Sequence generation	8a	Method used to generate the random allocation sequence	NA
	8b	Type of randomisation(s); details of any restriction (such as blocking and block size)	NA
Allocation concealment mechanism	9	Mechanism used to implement the random allocation sequence (such as sequentially numbered containers), describing any steps taken to conceal the sequence until interventions were assigned	NA

For peer review only

Implementation	10	Who generated the random allocation sequence, who enrolled participants, and who assigned participants to interventions	NA
Blinding	11a	If done, who was blinded after assignment to interventions (for example, participants, care providers, those assessing outcomes) and how	NA
	11b	If relevant, description of the similarity of interventions	NA
Statistical methods	12	Methods used to address each pilot trial objective whether qualitative or quantitative	10, 12
**Results**			
Participant flow (a diagram is strongly recommended)	13a	For each group, the numbers of participants who were approached and/or assessed for eligibility, randomly assigned, received intended treatment, and were assessed for each objective	NA (protocol)
	13b	For each group, losses and exclusions after randomisation, together with reasons	NA (protocol)
Recruitment	14a	Dates defining the periods of recruitment and follow-up	NA (protocol)
	14b	Why the pilot trial ended or was stopped	NA (protocol)
Baseline data	15	A table showing baseline demographic and clinical characteristics for each group	NA (protocol)
Numbers analysed	16	For each objective, number of participants (denominator) included in each analysis. If relevant, these numbers should be by randomised group	NA (protocol)
Outcomes and estimation	17	For each objective, results including expressions of uncertainty (such as 95% confidence interval) for any estimates. If relevant, these results should be by randomised group	NA (protocol)
Ancillary analyses	18	Results of any other analyses performed that could be used to inform the future definitive trial	NA (protocol)
Harms	19	All important harms or unintended effects in each group (for specific guidance see CONSORT for harms)	NA (protocol)
	19a	If relevant, other important unintended consequences	NA (protocol)
**Discussion**			
Limitations	20	Pilot trial limitations, addressing sources of potential bias and remaining uncertainty about feasibility	NA (protocol)
Generalisability	21	Generalisability (applicability) of pilot trial methods and findings to future definitive trial and other studies	16
Interpretation	22	Interpretation consistent with pilot trial objectives and findings, balancing potential benefits and harms, and considering other relevant evidence	NA (protocol)
	22a	Implications for progression from pilot to future definitive trial, including any proposed amendments	16
**Other information**			
Registration	23	Registration number for pilot trial and name of trial registry	2
Protocol	24	Where the pilot trial protocol can be accessed, if available	NA (protocol)
Funding	25	Sources of funding and other support (such as supply of drugs), role of funders	17
	26	Ethical approval or approval by research review committee, confirmed with reference number	16

For peer review only

Citation: Eldridge SM, Chan CL, Campbell MJ, Bond CM, Hopewell S, Thabane L, et al. CONSORT 2010 statement: extension to randomised pilot and feasibility trials. BMJ. 2016;355.
*We strongly recommend reading this statement in conjunction with the CONSORT 2010, extension to randomised pilot and feasibility trials, Explanation and Elaboration for important clarifications on all the items. If relevant, we also recommend reading CONSORT extensions for cluster randomised trials, non-inferiority and equivalence trials, non-pharmacological treatments, herbal interventions, and pragmatic trials. Additional extensions are forthcoming: for those and for up to date references relevant to this checklist, see www.consort-statement.org.

For peer review only
